# Single-Cell RNA Sequencing Reveals Cellular Heterogeneity and Stage Transition under Temperature Stress in Synchronized Plasmodium falciparum Cells

**DOI:** 10.1128/spectrum.00008-21

**Published:** 2021-07-07

**Authors:** Mukul Rawat, Ashish Srivastava, Shreya Johri, Ishaan Gupta, Krishanpal Karmodiya

**Affiliations:** a Department of Biology, Indian Institute of Science Education and Research, Pashan, Pune, Maharashtra, India; b Department of Biochemical Engineering and Biotechnology, Indian Institute of Technology Delhi, New Delhi, India; Broad Institute

**Keywords:** malaria, *Plasmodium falciparum*, antigenic variation, cellular heterogeneity, single-cell RNA sequencing, stress response, transcription

## Abstract

The malaria parasite has a complex life cycle exhibiting phenotypic and morphogenic variations in two different hosts by existing in heterogeneous developmental states. To investigate this cellular heterogeneity of the parasite within the human host, we performed single-cell RNA sequencing of synchronized *Plasmodium* cells under control and temperature treatment conditions. Using the Malaria Cell Atlas (https://www.sanger.ac.uk/science/tools/mca) as a guide, we identified 9 subtypes of the parasite distributed across known intraerythrocytic stages. Interestingly, temperature treatment results in the upregulation of the *AP2-G* gene, the master regulator of sexual development in a small subpopulation of the parasites. Moreover, we identified a heterogeneous stress-responsive subpopulation (clusters 5, 6, and 7 [∼10% of the total population]) that exhibits upregulation of stress response pathways under normal growth conditions. We also developed an online exploratory tool that will provide new insights into gene function under normal and temperature stress conditions. Thus, our study reveals important insights into cell-to-cell heterogeneity in the parasite population under temperature treatment that will be instrumental toward a mechanistic understanding of cellular adaptation and population dynamics in Plasmodium falciparum.

**IMPORTANCE** The malaria parasite has a complex life cycle exhibiting phenotypic variations in two different hosts accompanied by cell-to-cell variability that is important for stress tolerance, immune evasion, and drug resistance. To investigate cellular heterogeneity determined by gene expression, we performed single-cell RNA sequencing (scRNA-seq) of about 12,000 synchronized *Plasmodium* cells under physiologically relevant normal (37°C) and temperature stress (40°C) conditions phenocopying the cyclic bouts of fever experienced during malarial infection. In this study, we found that parasites exhibit transcriptional heterogeneity in an otherwise morphologically synchronized culture. Also, a subset of parasites is continually committed to gametocytogenesis and stress-responsive pathways. These observations have important implications for understanding the mechanisms of drug resistance generation and vaccine development against the malaria parasite.

## INTRODUCTION

Malaria is a major public health problem, with the parasite Plasmodium falciparum causing most of the malaria-associated mortality. Despite global efforts to eradicate the disease, hundreds of thousands of people die and around 200 million suffer due to malaria every year (1). P. falciparum completes its life cycle in two different hosts, progressing through multiple developmental stages. The persistent infection and clinical manifestation of malaria require development of the parasite in red blood cells (RBCs) via an asexual intraerythrocytic (IEC) developmental cycle (IDC). During the 48-h IDC, the parasite invades RBCs and develops into a ring stage, followed by trophozoite and schizont stages. The transmission of malaria parasites to mosquitoes depends on intraerythrocytic differentiation to gametocytes in a human host. Thus, parasites maintain a balance in transmission and persistent infection by an unknown mechanism. Recent single-cell RNA sequencing (scRNA-seq) studies suggest that parasites commit to gametocytes during the preceding cycles by epigenetic regulation of the master regulator of sexual development, the *AP2-G* gene (2–4). Also, Brancucci et al., showed that lysophosphatidylcholine metabolism regulates sexual differentiation in *Plasmodium* (5). However, the environmental cues and mechanism to initiate gametocytogenesis are hitherto unclear.

P. falciparum is exposed to various environmental conditions during its life cycle in two different hosts. Moreover, it is also constantly exposed to different physiological conditions, such as low glucose level (hypoglycemia) (6), high temperature (fever) (7), and oxidative stress (hemoglobin degradation and drug therapy) (8) during the IDC. Under such adverse conditions, some parasites in the isogenic population survive, whereas others fail, indicative of nongenetic sources of parasitic heterogeneity. The presence of heterogeneity is found in both unicellular and multicellular organisms (9, 10). Multicellular organisms are more advanced and developed in terms of fighting these fluctuations, where heterogeneity is the key to differentiation resulting in the diverse functions (11, 12). Heterogeneity through gene expression variegation serves as a population-level survival strategy in unicellular organisms (13, 14). Even slight changes in gene expression may affect the ability to survive stress and may confer competitive advantage over other individuals in a population. Consequently, ecological studies suggest that diverse populations have higher chances of survival during extreme conditions (9, 14–16). Therefore, understanding the cellular heterogeneity of *Plasmodium* in the context of stress could help elucidate mechanisms of adaptation and rampant emergence of drug resistance in the pathogen (14, 15).

Heat is one of the most common stresses experienced by *Plasmodium*. The release of merozoites during its IDC results in cyclic episodes of fever in the host, wherein the temperature rises as high as 41°C for about 2 to 6 h (17, 18). P. falciparum infection is known to induce high fever for a prolonged time (>24 h) (18). The early ring stage of parasite growth is more resistant, whereas later stages are sensitive to temperature treatment (19, 20). High temperature during malarial infection is known to be responsible for synchronizing *Plasmodium* growth in the human host (19). The stress response machinery is known to play an important role in establishment of successful infection inside the host. Previous studies on bulk populations of the parasite have identified various proteins that play an important role in temperature stress response by accelerating the growth and development of the parasite (20). Moreover, parasites exposed to higher temperature have increased expression of the virulence (*var*) gene, which enables the binding of parasite-infected RBCs to the endothelial receptors (CD36 and ICAM-1), leading to cytoadherence (20, 21). Recent reports also suggest that stress response is a crucial mediator of artemisinin resistance in P. falciparum (22–24).

*Plasmodium* is known to have tight gene regulation orchestrated at multiple levels of chromatin structure (25, 26), transcription (27–29), and translation (30, 31), which facilitates timely expression of different genes required at various stages of the parasite’s growth (32). Despite this tight regulation of gene expression, studies report transcriptional heterogeneity in the parasite populations (3, 33, 34). This cell-to-cell heterogeneity may be attributed to various environmental and physiological conditions, such as availability of nutrients, temperature fluctuations, cell cycle progression, and different morphological states. The advent of single-cell RNA sequencing (scRNA-seq) has been instrumental in unraveling the cellular heterogeneity within a bulk population of parasites (33, 35, 36). It provides information pertinent to different states coexisting in a cell population that lead to dynamic cell-to-cell variability in mRNA numbers and abundance. A recent study in *Plasmodium* using scRNA-seq profiled 165 single cells during IDC, revealed the transcriptional heterogeneity, and uncovered a gene signature for sexual stage in the parasite population (3). In this study, we have performed scRNA-seq under control and temperature treatment conditions to understand the change in cell-to-cell heterogeneity within and between the *Plasmodium* populations. Using this approach, we identified a combination of gene signatures that define cellular heterogeneity and stage transition during stress adaptation in the parasite. We propose that this redistribution may represent a generic model of how parasites react to different stress conditions. Thus, our scRNA-seq analysis suggests that the maintenance of cellular homeostasis should enable cells to survive under different stress conditions and may act as an important stimulator of development of drug resistance in Plasmodium falciparum.

## RESULTS

### Single-cell RNA sequencing during temperature treatment in P. falciparum.

**FIG 1:**
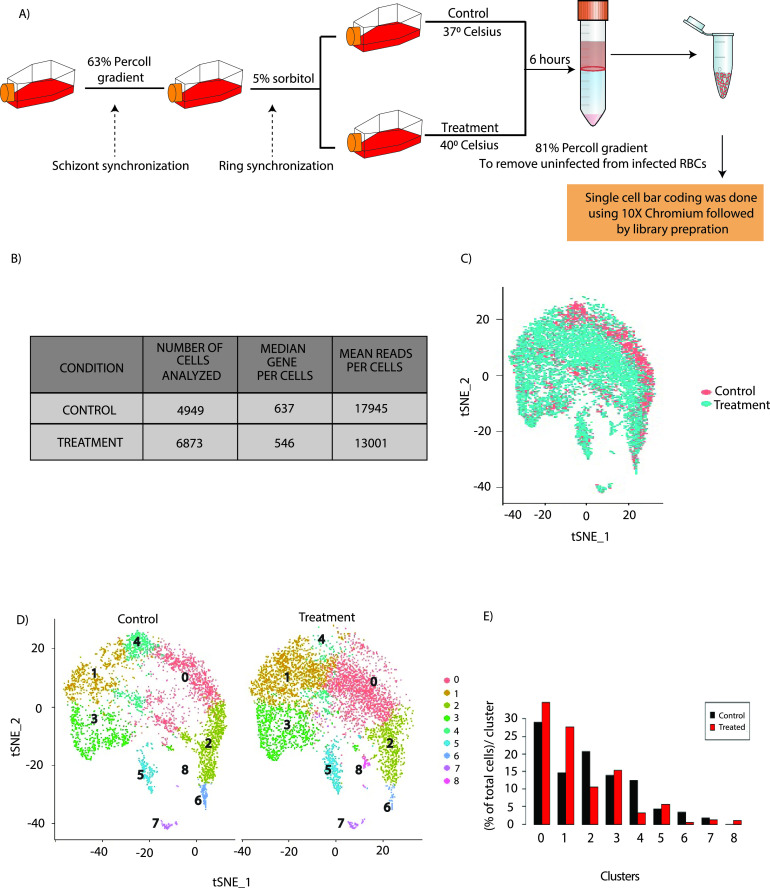
Single-cell RNA sequencing (scRNA-Seq) during control and temperature treatments in tightly synchronized P. falciparum parasites. (A) Schematic representing the pipeline used for stress induction, isolation of single parasites, and library preparation for scRNA-seq. Parasites were initially synchronized at the schizont stage using a 63% Percoll density gradient and later at the early ring stage using 5% sorbitol before starting the stress experiment. Stress was induced at 17 h postinvasion for a period of 6 h. Early trophozoite stage parasites were used for single-cell RNA sequencing. To separate uninfected from infected RBCs, an 81% Percoll density gradient (enriched infected early trophozoite stage) was used. Single-cell bar coding and library preparation were performed using standard 10× Chromium protocols with the Chromium Single-Cell 3′ reagent kit (v3) as per the manufacturer’s instruction. (B) Table indicating the number of single cells sequenced, median genes per cell, and mean reads identified per cell under control and temperature treatments. (C) t-SNE plots representing the combined clustering of cells sequenced under control (red) and temperature treatment (blue) conditions. (D) t-SNE plot representing the different clusters generated for the *Plasmodium* population under control and temperature treatment conditions. (E) Change in the percentage of cells in different clusters under temperature treatment.

*Plasmodium* harbored within human RBCs gets exposed to various environmental and physiological stresses. To understand the mechanism of stress tolerance and adaptation and the impact of these stresses on gene expression heterogeneity, we performed single-cell RNA sequencing (scRNA-seq) under the control condition and the physiological stress condition of high temperature (equivalent to fever) in P. falciparum. Parasites were synchronized at the schizont stage using a 63% Percoll density gradient and later at the early ring stage (0 to 3 h) using 5% sorbitol prior to the stress experiment. Temperature treatment was given at 40°C at the late ring stage (∼17 h) for a period of 6 h (Fig. 1A). Viability of the parasites is an important aspect to consider during febrile temperature for a longer period and at the later stages of the parasite growth. Therefore, in this study, we carefully selected the temperature (40°C), the time point (6 h of treatment), and the stage of the parasite (late-ring to early trophozoite stage), which have a minimal effect on parasite survival (Fig. 1A) (20, 37, 38). Moreover, we monitored the growth of the parasites using Giemsa stain upon temperature treatment, and we did not observe any significant change in the parasitemia after the 6-h temperature treatment as well in the following cycle (15 to 16% parasitemia) of IDC. Furthermore, we analyzed the parasites’ viability under control and temperature treatments using annexin V staining, which is commonly used to identify apoptotic parasites (39). Importantly, temperature treatment did not cause a significant change in the number of dead parasites, as assessed by fluorescence-activated cell sorter (FACS) analysis with fluorescein isothiocyanate (FITC)-annexin V staining (see Fig. S1A in the supplemental material). Before proceeding to single-cell library preparation, infected RBCs of control and treated parasites were enriched to 80% by using Percoll gradient centrifugation to remove uninfected RBCs. Enriched parasites under control and temperature-treated conditions were assayed for their single-cell gene expression profiles using the 10× Chromium single-cell RNA-sequencing v3 chemistry (Fig. 1A). We sequenced 4,949 single cells under the control condition and 6,873 single cells under the temperature treatment condition. The median numbers of genes detected per cell (count of ≥1) were 637 and 546, with sequencing depths (mean reads per cell per gene) of 17,945 and 13,001 for control and temperature-treated samples, respectively (Fig. 1B). Furthermore, in most published single-cell RNA sequencing data analyses, high mitochondrial gene expression is considered an indicator of cell death and is used as a cutoff to remove dead cells (40, 41). Therefore, to exclude the dead parasites from the analysis, we considered parasites (in both control and temperature treatments) that have a consistently low mitochondrial gene expression at less than 1% per cell. Furthermore, to remove potential doublets at a frequency of 5% and droplets with background RNA content, we removed cells with the top 5% and bottom 5% of total RNA molecules (or unique molecular identifiers [UMIs]) from each sample.

Elbow plots were generated, which identified control and the treated samples and showed that the percentage of variation explained per principal component (PC) flatlines by PC 10; therefore, 10 PCs were used downstream to perform t-SNE (*t*-distributed stochastic neighbor embedding) projections and clustering at a resolution of 0.5. Cells from both control and treatment samples were combined using canonical correlation analysis and clustered to identify transcriptionally distinct cells (Fig. 1C). As a result, we obtained 9 clusters labeled from “0” to “8” in the order of size, with “0” being the cluster with the largest number of cells (Fig. 1D). Furthermore, we identified marker genes specific to each cluster as being expressed in at least 30% of all the cells of the cluster with a 50% higher average expression than other clusters (see Fig. S1B and Table S1 in the supplemental material). Interestingly, we found a unique cluster (cluster 8) that emerged only during the temperature treatment (Fig. 1D). To confirm if cluster 8 represents a unique cluster or dead parasites, we analyzed mitochondrial gene expression in all the clusters (Fig. S1C) and found no significant difference in cluster 8. To further investigate if cluster 8 could have emerged due to a larger number of cells being assayed in the temperature treatment, we performed downsampling and were able to obtain cluster 8 (>30 cells) every time we performed clustering (Fig. S1D). Additionally, we performed random sampling and coclustering, and 97 out of 100 times, cells of the temperature-treated sample retained the unique cells of cluster 8 (Fig. S1E). Together, these findings suggest that cluster 8 is a unique cluster that emerged during temperature treatment. However, due to the absence of a more direct approach to differentiate live from dead cells using flow cytometry and the presence of a very small population in cluster 8, we do not exclude the possibility that cluster 8 includes some dead or dying cells. Moreover, significant changes in the numbers (percentages) of cells in different clusters (decrease in clusters 2, 4, and 6 and increase in cluster 1) during temperature treatment (Fig. 1E) are indicative of how the treatment rewired gene expression and biases the cells to exist in specific states that may be functionally relevant to the treatment.

### Assignment of developmental stages to clusters identifies heterogeneity within a synchronized population.

**FIG 2:**
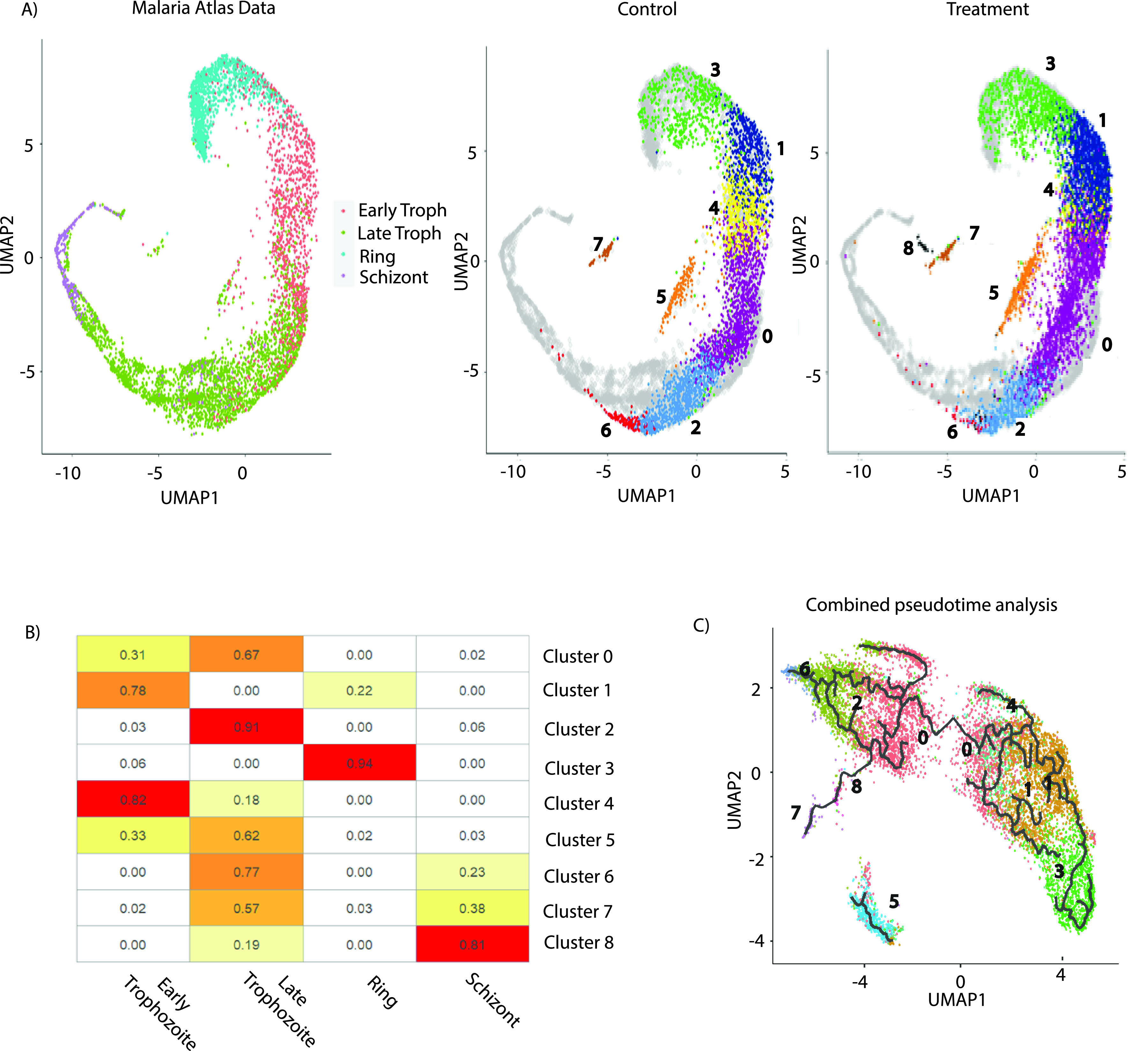
scRNA-seq reveals stage transitions. (A) Pseudotime analysis of the Malaria Cell Atlas (MCA) data with the assigned stages: ring, early trophozoite, late trophozoite, and schizont. Control and temperature-treated cells were coclustered with cells from MCA to identify the developmental stage of the clusters (identified in our study) based on the overlapping with MCA trajectory (shown in gray). (B) Heat map used to assign stages to clusters in our analysis, representing the percentage of cells from our clusters (rows) that cocluster with MCA data cells, with the assigned annotation per cell (columns). Cluster 3 coclusters most with ring stage parasites. Clusters 1, 4, and 0 (having only a few cells) cocluster with the early trophozoite stage. Clusters 0 (the majority of these cells), 2, 5, 6, and 7 cocluster with the late trophozoite stage, whereas cluster 8 coclusters with schizont cells from the MCA. (C) Pseudotime analysis of the parasites sequenced for a combined total of 11,822 of all the synchronized parasites (control and treatment together) sequenced under both control and temperature treatment conditions.

In order to further study the stage progression under the temperature treatment, we utilized single-cell data available from the Malaria Cell Atlas (42), which has annotation of cells at 4 bulk stages (ring, early trophozoite, late trophozoite, and schizont). The pseudotime analysis of the Malaria Cell Atlas also reflects the progression from ring to trophozoite to schizont stage through a continuing change in gene expression (Fig. 2A). Next, we coclustered our data from both control and temperature-treated *Plasmodium* cells and projected them onto the Malaria Cell Atlas pseudotime cell trajectory (shown as a translucent cloud of gray cells in the background in Fig. 2A) to assign names to various clusters. Surprisingly, our morphologically synchronized parasites exhibit significant transcriptional heterogeneity in both control and heat treatments, as depicted in the heat map (Fig. 2B). Next, we performed pseudotime analysis using the Monocle 3 package across the IDC of P. falciparum to understand finer details of the stage transition under temperature treatment. We performed pseudotime analysis for control and temperature-treated cells together (Fig. 2C). Since cluster 3 was identified as the ring stage (beginning of parasite developmental trajectory), it was chosen as the root for the pseudotime trajectory. As expected, we observed three transitions in pseudotime trajectory, when parasites move from cluster 3 to 1 (ring to early trophozoite), cluster 4 to 0 (early trophozoite to late trophozoite), and clusters 2 and 6 to 7 (late trophozoite to gametocyte), as parasites move from one stage of IDC to another (Fig. 2C). A separate pseudotime analysis for the control and heat treatment cells did not yield any additional information about the change in stage transition under temperature treatment. Thus, contrary to expectation, despite double synchronization of the parasites, we observed significant transcriptional heterogeneity and developmental variation under control and temperature treatment conditions. This is an important observation since most of the experiments performed in the field depend on the usual methods of synchronization, like the Percoll gradient and sorbitol treatment.

### Temperature treatment leads to decreased global transcription and increased transcriptional heterogeneity.

**FIG 3:**
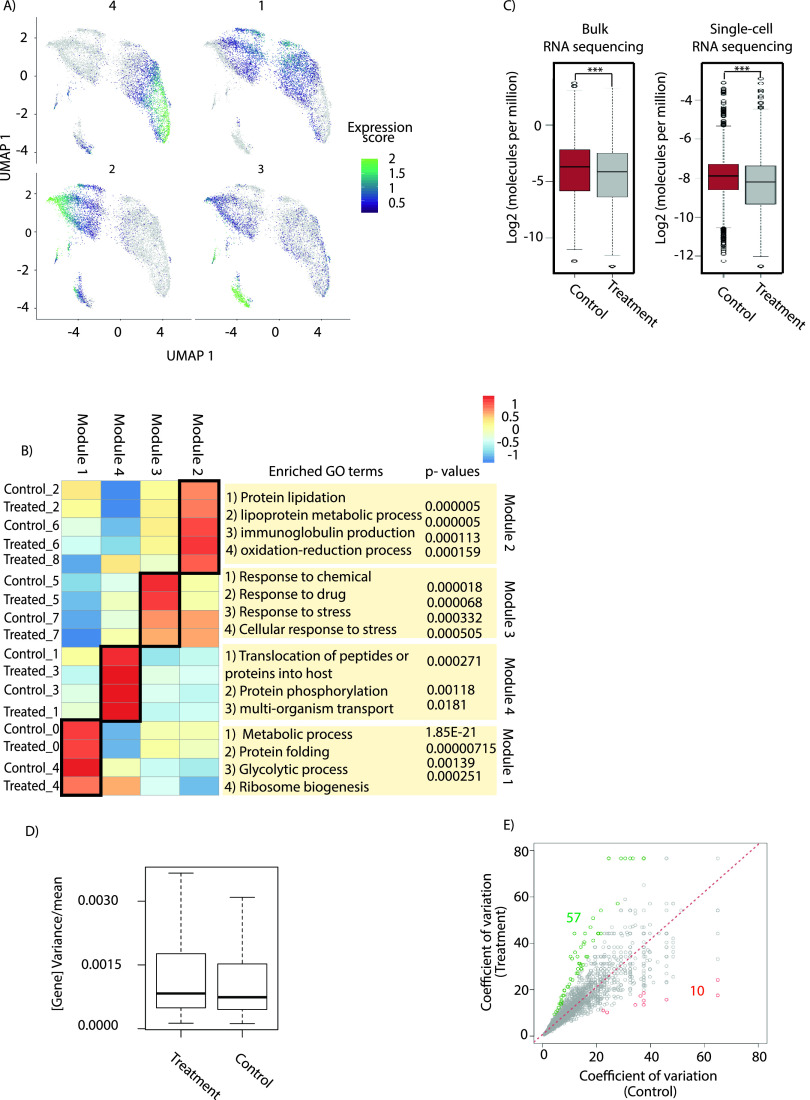
scRNA-seq reveals transcriptional heterogeneity during temperature treatment. (A) Genes were grouped into four modules (1 to 4) based on the similarity in their gene expression patterns. (B) Each module represents a unique set of genes that correspond to unique pathways (Gene Ontology terms) enriched in these different modules. Gene Ontology analysis was performed to identify these pathways/biological processes associated with each module. (C) Mean expression level of all the genes expressed during control and temperature treatment by both single-cell RNA sequencing and bulk RNA sequencing. Overall transcription is suppressed upon temperature treatment. Pseudo-bulked single-cell gene expression was correlated with gene expression under control (Spearman’s correlation, 0.51) and temperature treatment (Spearman’s correlation, 0.41) conditions. Significance was determined using a paired *t* test: ***, *P* < 0.005. (D) Comparison of the variance per mean (dispersion index) for all the genes between the control and temperature treatments. The dispersion is higher under temperature treatment compared to the control condition. The increase in gene expression variance under the temperature treatment is statistically significant, with a *P* value of 6.688e−10 (calculated using the Wilcoxon rank sum test). (E) Coefficients of variation are plotted for control and temperature treatments using scRNA-seq data. Under the control condition, only 10 genes were found with variation more than 2-fold, whereas under temperature treatment, 57 genes were found to show variation greater than 2-fold.

Since expression is regulated temporally in *Plasmodium* (28, 43), we performed gene expression network analysis and identified four modules (1 to 4) (Fig. 3A). Different modules correspond to the different developmental trajectory in the pseudotime analysis, which indicates the functional significance of these clusters based on transcriptional profile. Module 4 includes ring stage clusters, representing the initial stage of development, whereas module 1 represents early trophozoite clusters. Module 2 presents late trophozoite parasites, whereas module 3 contains clusters 5 and 7 (stress-responsive and gametocytogenesis-committed parasites, respectively). Moreover, Gene Ontology of different modules aligns with the Gene Ontology of the cluster-specific marker genes (Fig. 3B; see Fig. S2A and Table S2 in the supplemental material). Cluster 3, which shows a ring stage transcriptional profile, was found to be associated with transport of molecules into the host. Similarly, cluster 5 shows enrichment of “response to drug” and “response to stimulus,” which indicates that these parasites have expression of stress-responsive genes. Moreover, differential gene expression analysis across clusters during treatment identified cluster-specific deregulated pathways (Table S2). Interestingly, cluster 3 shows mutually exclusive up- and downregulation of genes important for the process of interaction with the hosts through adhesion (Fig. S2A). Stress-responsive cluster 5 shows upregulation of genes important in the DNA conformational change, which is known to affect replication and transcription (Fig. S2A).

Next, we looked at global transcriptional deregulation associated with the temperature treatment. Interestingly, we observed a global decrease in transcription in both scRNA-seq as well as bulk RNA sequencing under temperature treatment (Fig. 3C). Furthermore, to understand the cell-to-cell variability, we calculated the average number of RNA molecules per cell during control and temperature treatments. The average numbers of RNA molecules per cell were reduced during the temperature treatment, indicative of the fact that parasites slow down or shut off global transcription machinery during temperature treatment, resulting in delayed life cycle progression (Fig. S2B). The average numbers of RNA molecules are found to be decreased across the clusters, except cluster 1 (Fig. S2C). Furthermore, to validate this observation, we estimated the level of RNA molecules by labeling RNA using an RNA-specific dye, SYTO RNASelect, in control and treated samples. Flow cytometry analysis as well as confocal imaging showed a decrease in the level of RNA using SYTO RNASelect (Fig. S2D and S2E). Furthermore, to calculate the index of dispersion between the control and temperature-treated conditions, we plotted variance/mean or standard deviation/(*n* × mean) per gene from raw data. The increase in gene expression variance under the temperature treatment is statistically significant, with a *P* value of 6.688e−10 (calculated using the Wilcoxon rank sum test) (Fig. 3D). To understand the heterogeneity present between the control and temperature treatments, we calculated the measure of dispersion within a population using the coefficient of variation (CV) (Fig. 3E; Fig. S2F). We observed higher dispersion under temperature treatment for genes associated with gametocytogenesis, chaperone activity, and maintenance of cellular homeostasis (Fig. 3E; Fig. S2G).

### Temperature treatment results in upregulation of stress-responsive and regulators of gametocytogenesis genes.

**FIG 4:**
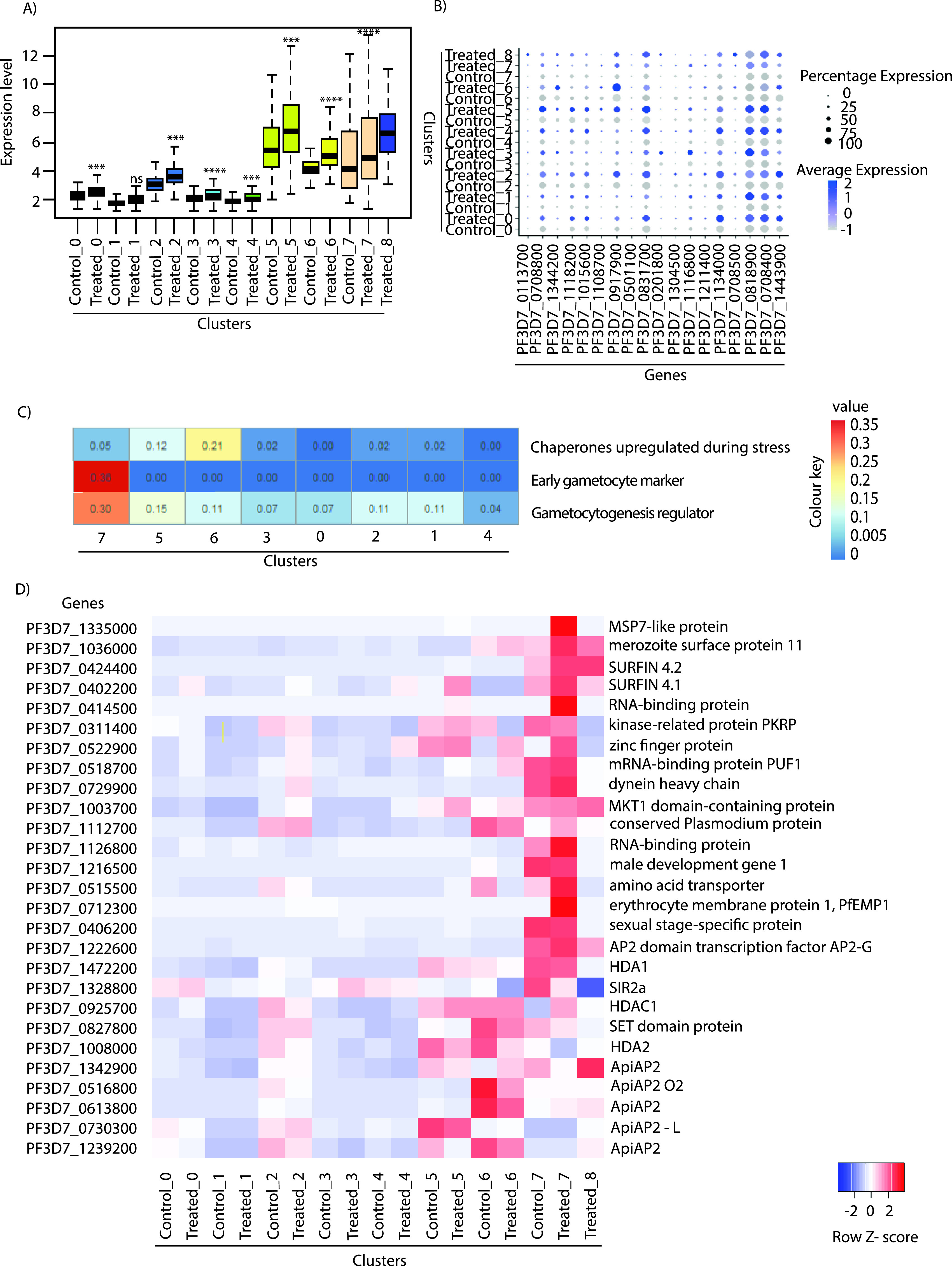
Gene expression analysis of stress-responsive genes under temperature treatment. (A) Comparison of the expression of stress-responsive genes in different clusters in control and temperature treatments. Gene expression is plotted over ribosomal proteins since expression of these genes was found to be relatively uniform in all of the clusters. Clusters 5, 6, and 7 show significant expression and upregulation of stress-responsive genes under temperature treatment. Significance was determined using a paired *t* test: ***, *P* < 0.001; ****, *P* < 0.0001. (B) A bubble plot shows relative upregulation of various heat shock proteins across clusters during temperature treatment as measured by scRNA sequencing. Bubble size is proportional to percentage of cells expressing a gene, and color intensity is proportional to average scaled gene expression within a cluster. (C) Heat map illustrating the relative expression level of chaperone proteins, different intraerythrocytic (IEC) stage markers, gametocyte markers, and gametocyte regulators across different clusters. A list of the genes used for generation of the heat map is provided in Table S5. (D) Heat map showing the relative expression of the AP2 transcription factors and different gametocytogenesis markers across clusters in the control and during temperature treatment. The cluster-specific enrichment of AP2 transcription factors indicates a distinct cluster-specific role of AP2 in gene expression programs. The colored scale bar at the bottom right side denotes the relative expression value, where −2 and 2 represent down- and upregulation of genes, respectively.

Most of the organisms have an evolutionarily conserved mechanism of stress response against a variety of environmental fluctuations. These stress response machineries consist of various proteins maintaining cellular homeostasis during unfavorable conditions (44, 45). To understand the transcriptional variation in stress-responsive genes under temperature treatment, we plotted expression levels of stress-responsive genes relative to ribosomal protein genes (which were found to be unchanged in all clusters). Though most of the clusters showed upregulation in the expression of stress-responsive genes, the clusters associated with stress and immune responses and gametocytogenesis (clusters 5 to 7) showed the highest overall expression and upregulation under temperature treatment (Fig. 4A; Fig. S3A). One of the most studied classes of proteins that play a crucial role in maintenance of cellular homeostasis is heat shock proteins (46–48). These proteins are known to play diverse functions like refolding of misfolded proteins and help with mRNA processing and maturation. We plotted expression of various heat shock proteins in each cluster and found them to be upregulated across the clusters upon temperature treatment (Fig. 4B). Similarly, we looked at the expression status of heat shock proteins (46) and ubiquitin proteasome pathway proteins (20) in bulk RNA sequencing under control and temperature-treated conditions. As expected, temperature treatment upregulates heat shock proteins and downregulates ubiquitin proteasome-related proteins (Fig. S3B). Moreover, marker genes from cluster 6 showed increased expression of different chaperone proteins that are known to play an important role during various stress responses (Fig. 4C).

Gametocytogenesis is a genetically encoded phenomenon, but it is known to be influenced and triggered by various environmental fluctuations (49, 50). Previous studies have suggested various factors such as host immune response, high parasitemia load, elevated temperature, drug treatment, etc., as triggers for the process of gametocytogenesis in P. falciparum (49–54). Furthermore, stress conditions are also shown to induce gametocytogenesis in *Plasmodium* (55, 56). In a similar context, we investigated the enrichment of gametocyte markers and regulators in various clusters under temperature treatment (Fig. 4C). Cluster-specific marker gene analysis showed that cluster 7 has higher expression of gametocyte marker genes, like the gamete antigen 27/25 gene (PF3D7_1302100), the sexual stage-specific protein precursor gene (PF3D7_0406200), the gametocyte erythrocyte cytosolic protein gene (PF3D7_1253000), the early gametocyte enriched phosphoprotein EGXP gene (PF3D7_1466200), and *GEXP02* (PF3D7_1102500) (57, 58), as well as the gametocytogenesis regulator, the AP2 domain transcription factor gene *AP2-G* (PF3D7_1222600) (3, 58, 59) (Table S1). Moreover, we also observed a significant overlap of our cluster 7 marker genes with recently identified markers of gametocytogenesis using scRNA-seq: the putative NYN domain-containing protein gene (PF3D7_0406500), the putative gene *CPSF* (cleavage and polyadenylation-specific factor) (PF3D7_0317700), the lysine-specific histone demethylase gene (PF3D7_0801900), and *HDA1* (PF3D7_1472200) (2, 3) (Table S1). Furthermore, many of the known gametocytogenesis marker genes like AP2 domain transcription factor gene *AP2-G*, the sexual stage-specific protein precursor gene, etc., were found to be further upregulated upon temperature stress (Fig. 4D; see Table S3 in the supplemental material). Thus, cluster 7 represents a rare population of parasites that are committed to gametocytogenesis, and exposure to higher temperature further upregulates the expression of some of the gametocytogenesis makers and regulators. Moreover, stress conditions like higher temperature and low doses of antimalarial drugs are shown to induce gametocytogenesis in P. falciparum (51, 54, 55, 60). This shows that temperature stress can upregulate the expression of gametocytogenesis regulators in a specific cluster. However, we have not experimentally demonstrated that temperature stress can affect the process of gametocytogenesis or sexual conversion rate.

Next, we explored if there is any specific transcription factor and epigenetic modifier associated with the regulation of stress/immune response and gametocytogenesis clusters. The ApiAp2 family transcription factors are known to regulate diverse biological processes in *Plasmodium* (58, 61–63). We identified an AP2 transcription factor (PF3D7_1239200) that is downregulated in cluster 5. Another AP2 transcription factor (PF3D7_1342900) was specifically upregulated in clusters 6, 7, and 8 during the temperature treatment (Fig. 4D). Other AP2 transcription factors with cluster-specific expression are represented in the heat map (Fig. 4D). Thus, it is plausible that these cluster-specific AP2 transcription factors may function as global/master regulators of respective clusters. Interestingly, we found the histone deacetylase gene *HDA1* showed increased expression in clusters 5, 6, and 7 (Fig. 4D). Furthermore, the expression of *HDA1* decreases upon temperature treatment, indicating that it may help in the upregulation of genes related to stress response and gametocytogenesis. Other histone modifier genes, such as *Sir2A*, *HDA2*, and the SET domain protein gene, were also found to be downregulated during temperature treatment in clusters 5, 6, and 7. Changes in the expression levels of these chromatin regulators indicate their possible role in the process of stress responses and gametocytogenesis.

### Export protein regulation plays an important role in stress response adaptation.

**FIG 5:**
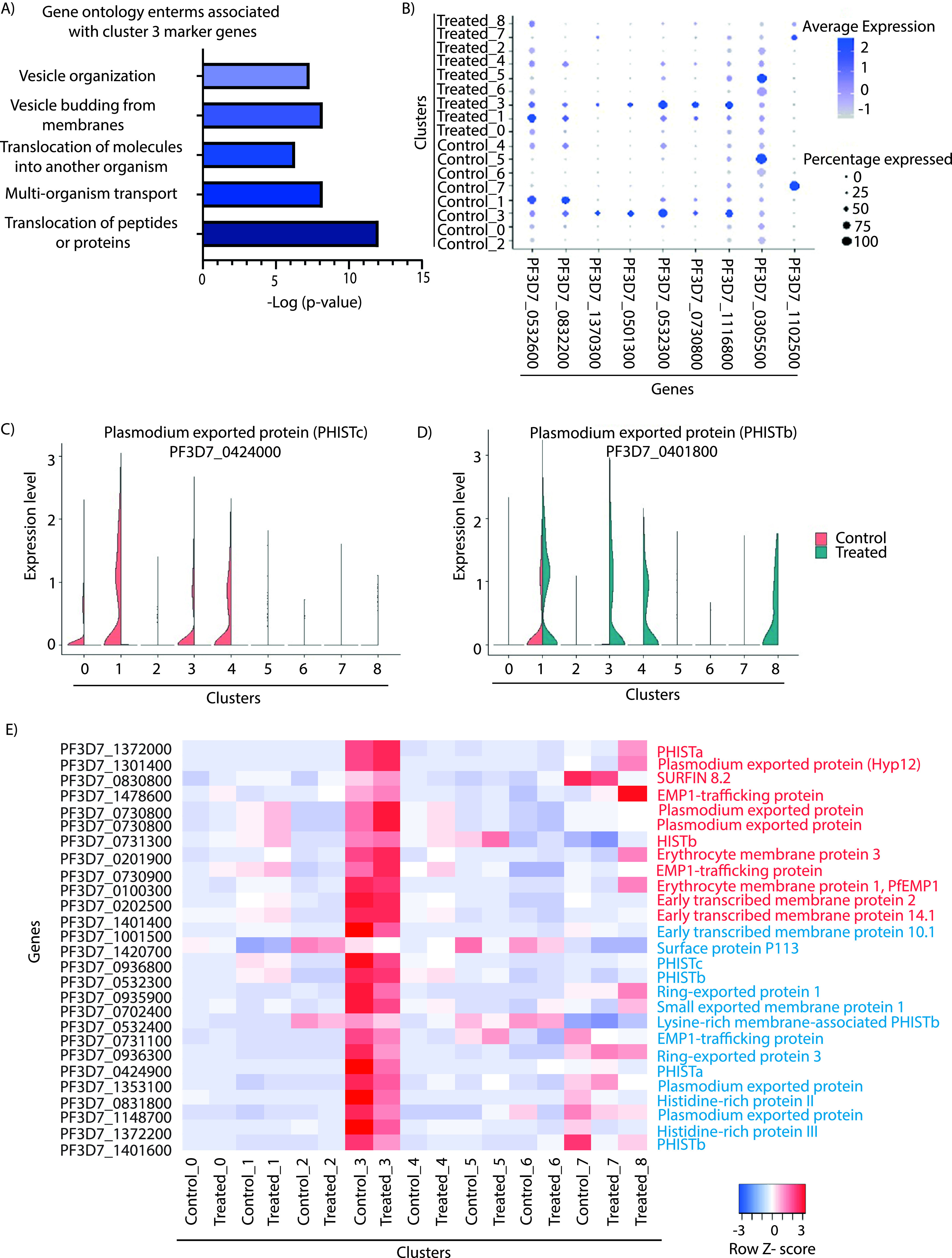
Expression of export proteins during temperature treatment. (A) Gene Ontology analysis of the marker genes of cluster 3. Pathways related to the transport of protein and peptides, translocation of molecules into other organisms, vesicles budding from the membrane, etc., were found to be enriched. (B) Bubble plot showing relative expression of transported proteins. Bubble size is proportional to percentage of cells expressing a gene, and color intensity is proportional to average scaled gene expression within a cluster. (C and D) Violin plots of representative examples of the transport-related proteins exhibiting mutually exclusive expression in control and temperature treatment across clusters. (E) Heat map showing the relative expression of transport proteins in the control and temperature treatment across clusters. Cluster 3 shows maximum expression of transport proteins. The colored scale bar at the bottom right side denotes the relative expression value, where −3 and 3 represent down- and upregulation of genes, respectively. Genes upregulated and downregulated under temperature treatment are represented by red and blue, respectively.

Previous reports have suggested the importance of export protein in initiating the process of sexual differentiation through host cell remodeling (64). Also, microarray studies have suggested a significant increase in the expression of transmembrane or secreted proteins during temperature treatment in P. falciparum (20). Interestingly, many of the exported proteins belong to the multivariant gene family, helping the parasites in sequestration by binding to various endothelial receptors (65, 66). Also, some export proteins mediate the process of rosetting, which helps in avoiding exposure of the parasite to the host immune response (67). Most of these proteins have either a PEXEL motif or host target signal sequence. Such transportation is usually mediated during the early stages of IDC (20). Though transported proteins are expressed in almost all clusters, they are significantly upregulated in cluster 3 (Fig. 5A and B). This is expected as most of the transportation-related processes take place during the ring stage (and cluster 3 also exhibits characteristics of ring stage parasites) (Fig. 2B). Some of the export proteins that are upregulated in cluster 3 include PfEMP1 (Plasmodium falciparum erythrocyte membrane protein 1) and a trafficking protein (which helps in the trafficking of PfEMP1 protein on the infected RBC surface), which mediates the virulence-associated changes over the *Plasmodium*-infected RBC surface (20). Intriguingly, cluster 3 also shows a mutually exclusive preference for transport proteins like PF3D7_0424000 (PHISTc) and PF3D7_0401800 (PHISTb) during temperature treatment (Fig. 5C and D). Various transport proteins showing deregulation during the temperature stress condition are represented in the heat map plotted for each cluster (Fig. 5E). Surprisingly, cluster 1 also shows overrepresentation of genes which help in transportation of proteins in parasites (Fig. 5B). Thus, cluster 1 might be the transition cluster, which originated from cluster 3, since parasites of these stages show expression of both ring and trophozoite stage markers and help in transportation of different proteins, as observed in the pseudotime analysis (Fig. 2C).

### Virulence gene regulation during the temperature stress condition.

Next, we decided to study the change in the transcription level of multivariant genes during temperature treatment. These genes’ products constitute a major fraction of the proteins transported from the parasites into the RBC membrane. Plasmodium falciparum carries several clonally variant multicopy gene families, such as *var*, *rifin*, and *stevor*, which are presented on the surface of infected RBCs upon expression. These proteins play a central role in enabling host immune evasion and promoting pathogenesis. PfEMP1 is the most expressed immunodominant antigen on infected RBCs (iRBCs). PfEMP1, encoded by a family of 60 *var* genes, is believed to exhibit antigenic switching upon immune exposure and/or environmental fluctuation and stress conditions (68–70). Bulk RNA sequencing studies have suggested expression of only one *var* gene in a population. However, the mechanism of selecting a single *var* gene for expression and suppressing the rest of the *var* genes is still not clear. A recent study using scRNA-seq employing Smart-seq2 indicated that the majority of the parasites (13 out of 17 individual cells) show expression of one dominant *var* gene, and only a few parasites express two *var* genes per cell (3).

**FIG 6:**
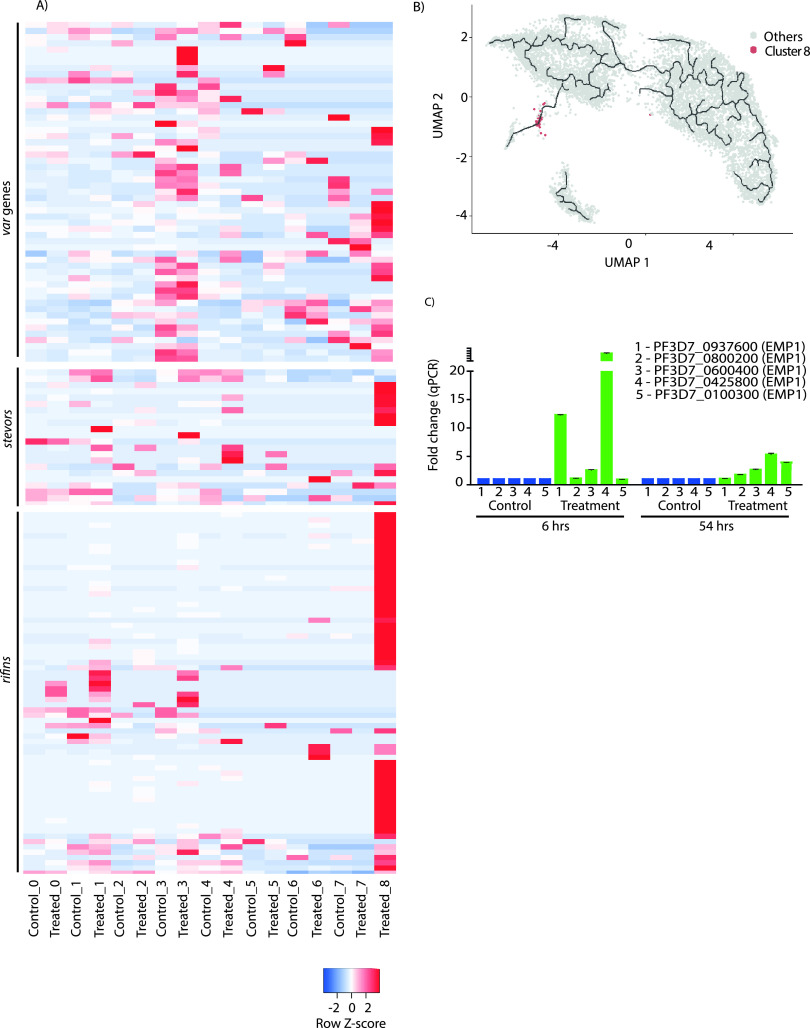
Expression of clonally variant multicopy gene families during temperature treatment. (A) Heat map showing the normalized expression of the *var*, *rifin*, and *stevor* genes under the control and temperature-treated conditions across clusters. The colored scale bar at bottom right side denotes the relative expression value, where −3 and 3 represent down- and upregulation of genes, respectively. Cluster 8, which is unique to temperature treatment, shows very high expression of clonally variant multicopy gene families. (B) Pseudotime analysis shows cluster 8 as the transitional cluster between cluster 7 (gametocytogenesis) and the rest of the parasite population. (C) RT-qPCR analysis of selected *var* genes under control and temperature treatments (after 6 h of treatment and 54 h after temperature treatment). The extended time point of 54 h (with parasites returned to 37°C after the 6-h temperature treatment) was used to investigate the effect of temperature treatment on *var* gene expression at a comparable time point during the next cycle of the parasite growth. *var* genes are upregulated upon 6 h of temperature treatment. Interestingly, even after 54 h of treatment, few *var* genes show slight upregulation in the expression level in comparison to the control.

We decided to look at the expression of *var* genes per cell using the scRNA sequencing under control and temperature treatments (Fig. 6A). Importantly, *var* genes are mostly expressed in cluster 3 (a transport-related cluster) and in cluster 8 (unique to temperature treatment). Moreover, cluster 8 exhibited exceptionally high expression of clonally variant multicopy gene families, such as *var*, *rifin*, and *stevor* (Fig. 6A), which play an important role in malaria pathogenesis by promoting cytoadherence, rosetting, and sequestration of the parasites (64, 65). Virulence of P. falciparum is associated with the cytoadhesion of the infected erythrocytes, leading to severe malaria (71–74). Since there is a positive correlation between *var* gene expression and pathogenicity, we believe cluster 8 is possibly a specific response of the pathogen against the elevated temperature. Furthermore, pseudotime analysis reveals that cluster 8, which is uniquely present during temperature treatment, is intermediate to the late trophozoite clusters 2, 6, and 7 (Fig. 6B). Moreover, cluster 8 was found to express the gametocyte marker AP2 domain transcription factor gene *AP2-G* (Fig. 4D), indicating that these parasites may convert into gametocytes if the stress is given for a longer duration.

Furthermore, to corroborate our findings, we investigated the expression levels of *var* genes in scRNA-seq and bulk RNA sequencing data under control and temperature-treated conditions. Interestingly, *var* genes are mostly upregulated in scRNA-seq as well as in bulk RNA sequencing under temperature treatment (Fig. S4A and S4B). This might also be because of the delayed progression in the life cycle of parasites under stress conditions. Since we found upregulation in the *var* gene at the transcript level, we decided to validate the deregulation of *var* genes using real-time quantitative PCR (RT-qPCR) after 6 h of treatment and 54 h after temperature treatment (Fig. 6C). The extended time point, 54 h (i.e., with parasites returned to 37°C after the 6-h temperature treatment), was used to investigate the effect of temperature stress on *var* gene expression at a comparable time point during the next cycle of the parasite growth. Interestingly, we found upregulation of two *var* genes at the 6- and 54-h time points (though to a lesser extent) after temperature treatment. This shows that febrile temperature can modulate the expression of *var* genes during the IDC for a few cycles.

## DISCUSSION

Parasites constantly face various environmental fluctuations during their life cycle, which can have different effects on parasite growth and development (75). Most organisms have a well-developed stress response machinery that helps them to cope up with the unfavorable conditions (76, 77). Such machinery is not well studied and characterized in P. falciparum. With recent studies appreciating the role of stress responses in drug resistance generation in P. falciparum (22, 23), it is important to look at the effect of stress conditions on cell-to-cell variability using single-cell RNA sequencing. Unlike previous scRNA-seq studies that were performed on mixed parasite populations (2, 42, 78) at different stages of growth, we performed scRNA sequencing of a larger number of cells at greater depth to better understand the composition and complexity of the synchronized *Plasmodium* culture. Furthermore, it is imperative to quantify transcriptional heterogeneity within synchronized cultures, as synchronization is performed on a regular basis for various *in vitro* experiments that are utilized to dissect the parasite’s biology in a controlled laboratory setting, and any effects due to underlying heterogeneity may bias the interpretation of these experiments.

We found nine transcriptionally distinct cell populations, all of which were represented in the Malaria Cell Atlas (42) (Fig. 2A). Thus, although parasites are morphologically synchronized, they exhibit a transcriptionally heterogeneous population. We also found significant changes in the numbers of cells in different clusters during temperature treatment (Fig. 1D and E). Change in the number of cells might be indicative of how the treatment rewired gene expression and biases the cells to exist in specific states that may be functionally relevant to the treatment (particularly evident for clusters 0 and 4). *In vivo*, the parasites are asynchronized during the initial stages of malaria infection within the human host. However, temperature stress (fever condition) occurring after every 48 h in the case of P. falciparum infection is known to synchronize these parasites eventually (79). This is in accordance with the previous observation that showed ring stage parasites are more resistant to stress conditions than other stages of the parasites (19, 20). Hence, under temperature treatment, the majority of the parasites slow down their progression—possibly to increase their survival chances.

Clusters 5, 6, and 7 represent about 10% of the parasites and are probably associated with stress adaptation under adverse conditions. Stress responsiveness is an important determinant for artemisinin drug resistance in P. falciparum (22, 23). Upregulation of stress-responsive pathways like unfolded protein response (UPR) and oxidative damage (redox stress responses) have been implicated in the mechanism of artemisinin resistance (22, 23). The importance of the stress-responsive pathway is evident from the fact that even a clonal population of resistant parasites does not show complete resistance (various rates by the ring survival assay [RSA], a measure of resistance against artemisinin) (80). Thus, this heterogeneous stress-responsive subpopulation (clusters 5, 6, and 7 [∼10% of the total population]), which exhibits upregulation of stress response pathways, might be associated with the possible artemisinin resistance in the parasites. Such findings would average out in bulk transcriptomic studies and may hamper the dissection of the mechanism of drug resistance in P. falciparum (22, 23). A subpopulation (cluster 7) of the parasites showed expression of various gametocyte markers and regulatory genes and thus looks committed for gametocytogenesis. Interestingly, this subpopulation does not change upon temperature exposure but upregulates expression of many gametocyte markers and regulators. Since lysophosphatidylcholine metabolism is known to trigger the process of gametocytogenesis, it will be interesting to see metabolic pathways that are modulated during heat stress in P. falciparum (5).

Thus, this study has identified the transcriptional heterogeneity in the parasite population and how such diversity in their cellular states responds discretely upon stress condition. Since several reports suggest the importance of stress response pathways in artemisinin resistance (22, 23), it is possible that genes showing maximum variation under stress conditions as well as drastic stage transitions under unfavorable conditions help cells to obtain a resistance-like phenotype. Importantly, the knowledge generated in this study could potentially be used to better understand the molecular mechanisms of drug resistance, pathogenesis, and virulence of the parasite.

### Limitations of the study.

In this study, we have provided important insights into the cell-to-cell heterogeneity under a temperature stress condition in a synchronized parasite population. Although we demonstrated that temperature stress upregulates the expression of gametocytogenesis regulators in a specific cluster, our work does not demonstrate the effect of temperature on either gametocytogenesis or sexual conversion rates. We also observed that temperature treatment results in the emergence of a novel cluster that shows higher expression of multivariant and stress-responsive genes. Unfortunately, due to technical limitations, we were not able to enrich the parasites of this cluster to understand their physiological significance. Subsequent work that utilizes stronger temperature treatment (either increasing the temperature or with treatment for longer duration) could add functional significance to our findings. These observations are clearly fertile ground for further exploration by the scientific community, and subsequent work will elucidate the specific effectors of these processes.

## MATERIALS AND METHODS

### *In vitro* culture of P. falciparum parasites and synchronization.

P. falciparum strain 3D7 cells were cultured in RPMI 1640 medium supplemented with 25 mM HEPES, 0.5% AlbuMAX II, 1.77 mM sodium bicarbonate, 100 μM hypoxanthine and 12.5 μg ml^−1^ gentamicin sulfate at 37°C. Parasites were maintained at 1 to 1.5% hematocrit and 5% parasitemia. Fresh O^+^ human RBCs were isolated from healthy human donors. Parasites were diluted after every 2 days by splitting the flask into 2 to 3 flasks in order to maintain the parasitemia around 5%. Parasites were synchronized at the schizont stage using a 63% Percoll density gradient and synchronized at the early ring stage using 5% sorbitol. For the stress experiment, parasites were kept at 8% parasitemia and 2% hematocrit. Parasite growth was monitored using Giemsa staining of a thin blood smear. Parasites were maintained in an incubator with 5% CO_2_. Annexin V-FITC (ab14085; Abcam) staining was performed as mentioned in the protocol.

### FACS analysis and confocal imaging.

Parasites were stained with annexin V for 10 min in a binding buffer before proceeding to flow cytometry. Parasites were stained using SYTO RNASelect (Thermo) for 30 min in RPMI with 0.5% fetal bovine serum (FBS). Parasites were washed and resuspended in 1× phosphate-buffered saline (PBS) before proceeding to FACS analysis. Flow cytometry analysis was performed using BD FACS Aria Fusion (BD Biosciences), and data were analyzed using FlowJo software (www.flowgo.com). Stained parasites were also smeared on glass slides for confocal imaging. Parasites were fixed with methanol and mounted with Prolong Gold antifade with DAPI (4′,6-diamidino-2-phenylindole) (Thermo). Confocal images were acquired using Leica SP8 microscope (Leica).

### Stress induction.

Tightly synchronized parasites were given a temperature treatment of 40°C for 6 h from the late ring stage (∼17 h) to the early trophozoite stage (∼23 h). Control parasites were kept under normal culture conditions (37°C).

### Percoll gradient and isolation of single iRBCs.

In order to get rid of the uninfected parasites, we performed a Percoll gradient centrifugation to separate uninfected from infected RBCs. Different percentages of the Percoll gradient were tried for separation of early trophozoites from uninfected parasites. A 63% Percoll gradient was used for synchronization to separate schizonts from uninfected parasites. An 81% Percoll gradient was standardized and finally used for the enrichment of the infected parasites after the experiment. Parasites were washed with 1× PBS twice to get rid of any dead parasites, and parasites were counted before barcoding of the cells.

### scRNA-seq library preparation.

Gel beads in emulsion (GEMs) are generated by combining barcoded Single-Cell 3′ v3 gel beads, a master mixture containing cells and partitioning oil, onto Chromium Chip B. Cells are delivered at a limiting dilution in order to attain single-cell resolution. Once the gel beads are dissolved and cells are lysed inside the oil droplet, primers are released, which bind to the poly(A) tail of the mRNAs and help with the cDNA synthesis. Primers contain an Illumina TruSeq Read 1, 16-nucleotide 10× barcode, 12-nucleotide unique molecular identifier (UMI), and 30-nucleotide poly(dT) sequence. Enzymatic fragmentation and size selection were used to optimize the cDNA amplicon.

### scRNA-seq at NextSeq550 (Illumina).

Quality of the cDNA was estimated using a Bioanalyzer, and the concentration of the sample was calculated using HS DNA Qubit. Transcriptome sequencing was performed using the Illumina NextSeq 550 system (2 × 75-bp read length) at the NGS Facility, IISER, Pune, India.

### RNA sequencing and data analysis.

Bulk RNA sequencing was performed using parasites harvested for RNA isolation after the stress induction. Total RNA isolation was performed using TRIzol reagent. The Bioanalyzer was used to analyze the quality of RNA before proceeding to library preparation. Three biological replicates were pooled for RNA sequencing. The cDNA libraries were prepared for samples using the Agilent SureSelect strand-specific RNA library preparation kit. Transcriptome sequencing was performed using the Illumina NextSeq 550 system (2 × 150-bp read length) at the NGS Facility, IISER, Pune. Quality control of the RNA sequencing reads was performed using FASTQC, and reads were trimmed based on the quality estimates. RNA paired directional reads were mapped to GTF annotation file v41.0 of the P. falciparum genome using TopHat. SAMtools (81) were used for file handling and conversion. Cufflinks programs (cuffmerge, cuffmerge, and cuffdiff) (82) were used for differential gene expression. The MA plot was generated using R software (http://r-project.org/). Gene Ontology was performed using Plasmodb (83; https://plasmodb.org/).

### scRNA-seq library preparation and sequencing.

A 1,000-cell/μl dilution of 80% enriched infected parasites was loaded onto the 10× chip for library preparation using Single-cell v3 chemistry. The libraries for both the control and temperature-treated samples were multiplexed together and were sequenced using a mid-output 75-bp single-end-configuration flow cell on the Illumina NextSeq 550 system. Cell Ranger count was run on samples using v41.0 P. falciparum genome (PF3D7) to align and summarize per-cell barcode (cell) read counts for each sample. For control samples, 4,949 cells were sequenced at a depth of 88.8 million reads, with 88.1% reads in cells, assigning a median of 17,900 reads/cell. For the treated sample, 6,873 cells were sequenced at a depth of 89.3 million reads, with 88% of reads in cells, assigning a median of 13,000 reads/cell.

### scRNA-sequencing quality control and data analysis.

The sparse matrices generated by 10× cell ranger count were read into R 3.5.3 using the Seurat 3.0.0 package. In order to remove potential doublets at a frequency of 5% and droplets with background RNA content, we removed cells with the top 5% and bottom 5% of total RNA molecules (or UMIs) from each sample. Thereafter, both of the samples were combined using the canonical correlation analysis with 10 principal components to find integration anchors followed by 20 principal components to integrate the data. Twenty principal components were used to perform t-SNE projections and clustering at a resolution of 0.5 using the Find Neighbours and Find Clusters functions (Fig. S5). Cluster-specific markers were identified using the Find Conserved Markers function (with default parameters), and differentially expressed genes were identified using the Find Markers function (with default parameters). Pseudotime analysis was performed using the Monocle 3 package, assuming a binomial distribution with a detection limit of 0.5 and a minimum normalized gene expression of 0.1. The marker genes for different clusters obtained from Seurat analysis described above were used to calculate pseudotime and order the cells along the pseudotime trajectory.

### Data analysis.

GraphPad (www.graphpad.com) was used for plotting the normalized log_2_ expression ratios of the RNA sample/3D7 RNA reference pool for all probes. Single-cell data were obtained from The Malaria Cell Atlas (https://www.sanger.ac.uk/science/tools/mca).

### Primer design and RT-qPCR analysis.

Total RNA was isolated from the parasites using TRIzol reagent (Bio-Rad). Post-DNase treatment, 2 μg RNA was used for cDNA synthesis with the ImProm-II reverse transcription system (Promega), as per the manufacturer's recommendation. Real-time PCR was carried out using a CFX96 real-time PCR detection system (Bio-Rad). 18S rRNA and tRNA synthetase were used as an internal control to normalize for variability across different samples. Quantification of the expression was done with the help of fluorescence readout of SYBR green dye incorporation into the amplifying targets (Bio-Rad). Each experiment included technical replicates and was performed over three independent biological replicates. The primers used for real-time quantitative PCR (RT-qPCR) are listed in Table S4 in the supplemental material.

### Fligner-Killeen test.

The coefficient of variation (CV) was calculated as the standard deviation/mean for 5,066 P. falciparum genes individually and plotted. The coefficient of variation of individual genes was used to calculate the coefficient of variation for the control and temperature-treated conditions using Past3 (https://www.nhm.uio.no/english/research/infrastructure/past/). The Fligner-Killeen Test (a nonparametric test for homogeneity of group variances based on ranks) for equal coefficients of variation suggested significant variation between control and stress conditions (CVs of 128.19 and 136.32 for control and temperature treatments, respectively; *P* = 2.08e−5).

### Interactive web application.

An interactive web application and visualization were created using the open package from Rstudio called “Shiny apps” (https://www.shinyapps.io/). The interactive tool (iitd.info/synchronised_plasmodium_cell_atlas/) allowed easy visualization of the data set of single-cell sequencing performed under control and temperature treatment conditions. Expression of a particular gene (violin plots) and t-SNE projection of a particular gene could be generated by entering the *Plasmodium* gene ID. Pseudotime analysis of control and treatment samples for different cells sequenced could be also analyzed. Furthermore, we have also incorporated Malaria Cell Atlas data for parallel comparison with our sequenced data.

### Ethics statement.

This study did not involve human participants. The human RBCs used in this study were obtained from the KEM Blood Bank (Pune, India) as blood from anonymized donors. Approval to use this material for P. falciparum *in vitro* culture has been granted by the Institutional Biosafety Committee of the Indian Institute of Science Education and Research Pune (BT/BS/17/582/2014-PID).

### Data availability.

Further information and requests for resources and reagents should be directed to and will be fulfilled by the lead contact, Krishanpal Karmodiya (krish@iiserpune.ac.in). Note that this study did not generate new unique reagents. Single-cell RNA sequencing data for *Plasmodium* for the control and temperature treatments have been submitted to the Sequence Read Archive (SRA) under accession no. PRJNA560557.

## References

[1] World Health Organization. 2019. World malaria report 2019. https://www.who.int/publications/i/item/9789241565721.

[2] Poran A, Nötzel C, Aly O, Mencia-Trinchant N, Harris CT, Guzman ML, Hassane DC, Elemento O, Kafsack BF. 2017. Single-cell RNA sequencing reveals a signature of sexual commitment in malaria parasites. Nature 551:95–99. doi:10.1038/nature24280. 29094698 PMC6055935

[3] Ngara M, Palmkvist M, Sagasser S, Hjelmqvist D, Björklund ÅK, Wahlgren M, Ankarklev J, Sandberg R. 2018. Exploring parasite heterogeneity using single-cell RNA-seq reveals a gene signature among sexual stage Plasmodium falciparum parasites. Exp Cell Res 371:130–138. doi:10.1016/j.yexcr.2018.08.003. 30096287

[4] Reid AJ, Talman AM, Bennett HM, Gomes AR, Sanders MJ, Illingworth CJ, Billker O, Berriman M, Lawniczak MK. 2018. Single-cell RNA-seq reveals hidden transcriptional variation in malaria parasites. eLife 7:e33105. doi:10.7554/eLife.33105. 29580379 PMC5871331

[5] Brancucci NMB, Gerdt JP, Wang CQi, De Niz M, Philip N, Adapa SR, Zhang M, Hitz E, Niederwieser I, Boltryk SD, Laffitte M-C, Clark MA, Grüring C, Ravel D, Blancke Soares A, Demas A, Bopp S, Rubio-Ruiz B, Conejo-Garcia A, Wirth DF, Gendaszewska-Darmach E, Duraisingh MT, Adams JH, Voss TS, Waters AP, Jiang RHY, Clardy J, Marti M. 2017. Lysophosphatidylcholine regulates sexual stage differentiation in the human malaria parasite Plasmodium falciparum. Cell 171:1532–1544.e15. doi:10.1016/j.cell.2017.10.020. 29129376 PMC5733390

[6] Thien HV, Kager PA, Sauerwein HP. 2006. Hypoglycemia in falciparum malaria: is fasting an unrecognized and insufficiently emphasized risk factor? Trends Parasitol 22:410–415. doi:10.1016/j.pt.2006.06.014. 16839817

[7] Pavithra SR, Banumathy G, Joy O, Singh V, Tatu U. 2004. Recurrent fever promotes Plasmodium falciparum development in human erythrocytes. J Biol Chem 279:46692–46699. doi:10.1074/jbc.M409165200. 15339915

[8] Percário S, Moreira DR, Gomes BA, Ferreira ME, Gonçalves ACM, Laurindo PS, Vilhena TC, Dolabela MF, Green MD. 2012. Oxidative stress in malaria. Int J Mol Sci 13:16346–16372. doi:10.3390/ijms131216346. 23208374 PMC3546694

[9] Patra P, Klumpp S. 2014. Phenotypically heterogeneous populations in spatially heterogeneous environments. Phys Rev E 89:e030702. doi:10.1103/PhysRevE.89.030702. 24730780

[10] Altschuler SJ, Wu LF. 2010. Cellular heterogeneity: do differences make a difference? Cell 141:559–563. doi:10.1016/j.cell.2010.04.033. 20478246 PMC2918286

[11] Smith S, Grima R. 2018. Single-cell variability in multicellular organisms. Nat Commun 9:345. doi:10.1038/s41467-017-02710-x. 29367605 PMC5783944

[12] Brock A, Chang H, Huang S. 2009. Non-genetic heterogeneity—a mutation-independent driving force for the somatic evolution of tumours. Nat Rev Genet 10:336–342. doi:10.1038/nrg2556. 19337290

[13] Martins BM, Locke JC. 2015. Microbial individuality: how single-cell heterogeneity enables population level strategies. Curr Opin Microbiol 24:104–112. doi:10.1016/j.mib.2015.01.003. 25662921

[14] Goldman SL, MacKay MJ, Afshinnekoo E, Melnick A, Wu S, Mason CE. 2019. The impact of heterogeneity on single-cell sequencing. Front Genet 10:8. doi:10.3389/fgene.2019.00008. 30881372 PMC6405636

[15] Chisholm RH, Lorenzi T, Clairambault J. 2016. Cell population heterogeneity and evolution towards drug resistance in cancer: biological and mathematical assessment, theoretical treatment optimisation. Biochim Biophys Acta 1860:2627–2645. doi:10.1016/j.bbagen.2016.06.009. 27339473

[16] Starrfelt J, Kokko H. 2012. Bet‐hedging—a triple trade‐off between means, variances and correlations. Biol Rev 87:742–755. doi:10.1111/j.1469-185X.2012.00225.x. 22404978

[17] Kitchen S. 1949. Symptomatology: general considerations. Malariology 2:966–994.

[18] Karunaweera ND, Grau GE, Gamage P, Carter R, Mendis KN. 1992. Dynamics of fever and serum levels of tumor necrosis factor are closely associated during clinical paroxysms in Plasmodium vivax malaria. Proc Natl Acad Sci U S A 89:3200–3203. doi:10.1073/pnas.89.8.3200. 1565611 PMC48833

[19] Kwiatkowski D. 1989. Febrile temperatures can synchronize the growth of Plasmodium falciparum in vitro. J Exp Med 169:357–361. doi:10.1084/jem.169.1.357. 2642531 PMC2189185

[20] Oakley MS, Kumar S, Anantharaman V, Zheng H, Mahajan B, Haynes JD, Moch JK, Fairhurst R, McCutchan TF, Aravind L. 2007. Molecular factors and biochemical pathways induced by febrile temperature in intraerythrocytic Plasmodium falciparum parasites. Infect Immun 75:2012–2025. doi:10.1128/IAI.01236-06. 17283083 PMC1865691

[21] Udomsangpetch R, Pipitaporn B, Silamut K, Pinches R, Kyes S, Looareesuwan S, Newbold C, White NJ. 2002. Febrile temperatures induce cytoadherence of ring-stage Plasmodium falciparum-infected erythrocytes. Proc Natl Acad Sci U S A 99:11825–11829. doi:10.1073/pnas.172398999. 12177447 PMC129353

[22] Rocamora F, Zhu L, Liong KY, Dondorp A, Miotto O, Mok S, Bozdech Z. 2018. Oxidative stress and protein damage responses mediate artemisinin resistance in malaria parasites. PLoS Pathog 14:e1006930. doi:10.1371/journal.ppat.1006930. 29538461 PMC5868857

[23] Dogovski C, Xie SC, Burgio G, Bridgford J, Mok S, McCaw JM, Chotivanich K, Kenny S, Gnädig N, Straimer J, Bozdech Z, Fidock DA, Simpson JA, Dondorp AM, Foote S, Klonis N, Tilley L. 2015. Targeting the cell stress response of Plasmodium falciparum to overcome artemisinin resistance. PLoS Biol 13:e1002132. doi:10.1371/journal.pbio.1002132. 25901609 PMC4406523

[24] Tilley L, Straimer J, Gnädig NF, Ralph SA, Fidock DA. 2016. Artemisinin action and resistance in Plasmodium falciparum. Trends Parasitol 32:682–696. doi:10.1016/j.pt.2016.05.010. 27289273 PMC5007624

[25] Abel S, Le Roch KG. 2019. The role of epigenetics and chromatin structure in transcriptional regulation in malaria parasites. Brief Funct Genomics 18:302–313. doi:10.1093/bfgp/elz005. 31220857 PMC6859822

[26] Batugedara G, Lu XM, Bunnik EM, Le Roch KG. 2017. The role of chromatin structure in gene regulation of the human malaria parasite. Trends Parasitol 33:364–377. doi:10.1016/j.pt.2016.12.004. 28065669 PMC5410391

[27] Karmodiya K, Pradhan SJ, Joshi B, Jangid R, Reddy PC, Galande S. 2015. A comprehensive epigenome map of Plasmodium falciparum reveals unique mechanisms of transcriptional regulation and identifies H3K36me2 as a global mark of gene suppression. Epigenetics Chromatin 8:32. doi:10.1186/s13072-015-0029-1. 26388940 PMC4574195

[28] Gupta AP, Chin WH, Zhu L, Mok S, Luah Y-H, Lim E-H, Bozdech Z. 2013. Dynamic epigenetic regulation of gene expression during the life cycle of malaria parasite Plasmodium falciparum. PLoS Pathog 9:e1003170. doi:10.1371/journal.ppat.1003170. 23468622 PMC3585154

[29] Coleman BI, Duraisingh MT. 2008. Transcriptional control and gene silencing in Plasmodium falciparum. Cell Microbiol 10:1935–1946. doi:10.1111/j.1462-5822.2008.01203.x. 18637022 PMC12801279

[30] Cui L, Lindner S, Miao J. 2015. Translational regulation during stage transitions in malaria parasites. Ann N Y Acad Sci 1342:1–9. doi:10.1111/nyas.12573. 25387887 PMC4405408

[31] Vembar SS, Droll D, Scherf A. 2016. Translational regulation in blood stages of the malaria parasite Plasmodium spp.: systems‐wide studies pave the way. WIREs RNA 7:772–792. doi:10.1002/wrna.1365. 27230797 PMC5111744

[32] Foth BJ, Zhang N, Chaal BK, Sze SK, Preiser PR, Bozdech Z. 2011. Quantitative time-course profiling of parasite and host cell proteins in the human malaria parasite Plasmodium falciparum. Mol Cell Proteomics 10:M110.006411. doi:10.1074/mcp.M110.006411. PMC3149090 21558492

[33] Choi YH, Kim JK. 2019. Dissecting cellular heterogeneity using single-cell RNA sequencing. Mol Cells 42:189–199. doi:10.14348/molcells.2019.2446. 30764602 PMC6449718

[34] Rovira-Graells N, Gupta AP, Planet E, Crowley VM, Mok S, de Pouplana LR, Preiser PR, Bozdech Z, Cortés A. 2012. Transcriptional variation in the malaria parasite Plasmodium falciparum. Genome Res 22:925–938. doi:10.1101/gr.129692.111. 22415456 PMC3337437

[35] Peng J, Sun B-F, Chen C-Y, Zhou J-Y, Chen Y-S, Chen H, Liu L, Huang D, Jiang J, Cui G-S, Yang Y, Wang W, Guo D, Dai M, Guo J, Zhang T, Liao Q, Liu Y, Zhao Y-L, Han D-L, Zhao Y, Yang Y-G, Wu W. 2019. Single-cell RNA-seq highlights intra-tumoral heterogeneity and malignant progression in pancreatic ductal adenocarcinoma. Cell Res 29:725–738. doi:10.1038/s41422-019-0195-y. 31273297 PMC6796938

[36] Cheng S, Pei Y, He L, Peng G, Reinius B, Tam PP, Jing N, Deng Q. 2019. Single-cell RNA-seq reveals cellular heterogeneity of pluripotency transition and x chromosome dynamics during early mouse development. Cell Rep 26:2593–2607.e3. doi:10.1016/j.celrep.2019.02.031. 30840884

[37] Lu K-Y, Pasaje CFA, Srivastava T, Loiselle DR, Niles J, Derbyshire E. 2020. Phosphatidylinositol 3-phosphate and Hsp70 protect Plasmodium falciparum from heat-induced cell death. bioRxiv. https://www.biorxiv.org/content/10.1101/2020.03.17.995050v1. 10.7554/eLife.56773 PMC7518890 32975513

[38] Aunpad R, Somsri S, Na-Bangchang K, Udomsangpetch R, Mungthin M, Adisakwattana P, Chaijaroenkul W. 2009. The effect of mimicking febrile temperature and drug stress on malarial development. Ann Clin Microbiol Antimicrob 8:19. doi:10.1186/1476-0711-8-19. 19523215 PMC2707362

[39] Totino PRR, das Dores Magalhães A, Alves EB, Costa MRF, de Lacerda MVG, Daniel-Ribeiro CT, Ferreira-da-Cruz MdF. 2014. Plasmodium falciparum, but not P vivax, can induce erythrocytic apoptosis. Parasit Vectors 7:484. doi:10.1186/s13071-014-0484-8. 25325923 PMC4206708

[40] Tomoiaga D, Aguiar-Pulido V, Shrestha S, Feinstein P, Levy SE, Mason CE, Rosenfeld JA. 2020. Single-cell sperm transcriptomes and variants from fathers of children with and without autism spectrum disorder. NPJ Genom Med 5:7. doi:10.1038/s41525-020-0117-4. 32133155 PMC7035312

[41] Liao J, Yu Z, Chen Y, Bao M, Zou C, Zhang H, Liu D, Li T, Zhang Q, Li J, Cheng J, Mo Z. 2020. Single-cell RNA sequencing of human kidney. Sci Data 7:9. doi:10.1038/s41597-019-0351-8. 31896769 PMC6940381

[42] Howick VM, Russell AJC, Andrews T, Heaton H, Reid AJ, Natarajan K, Butungi H, Metcalf T, Verzier LH, Rayner JC, Berriman M, Herren JK, Billker O, Hemberg M, Talman AM, Lawniczak MKN. 2019. The Malaria Cell Atlas: single parasite transcriptomes across the complete Plasmodium life cycle. Science 365:eaaw2619. doi:10.1126/science.aaw2619. 31439762 PMC7056351

[43] de Azevedo MF, del Portillo HA. 2007. Control of gene expression in Plasmodium. Chapter B01. *In* Gruber A, Durham AM, Huyhn C, del Portillo HA (ed), Bioinformatics in tropical disease research: a practical and case-study approach. National Center for Biotechnology Information, Bethesda, MD.

[44] Galluzzi L, Yamazaki T, Kroemer G. 2018. Linking cellular stress responses to systemic homeostasis. Nat Rev Mol Cell Biol 19:731–745. doi:10.1038/s41580-018-0068-0. 30305710

[45] Stacchiotti A. 2019. Exploring cellular stress response and chaperones. Cells 8:408. doi:10.3390/cells8050408. 31052568 PMC6562650

[46] Jacob P, Hirt H, Bendahmane A. 2017. The heat‐shock protein/chaperone network and multiple stress resistance. Plant Biotechnol J 15:405–414. doi:10.1111/pbi.12659. 27860233 PMC5362687

[47] Feder ME, Hofmann GE. 1999. Heat-shock proteins, molecular chaperones, and the stress response: evolutionary and ecological physiology. Annu Rev Physiol 61:243–282. doi:10.1146/annurev.physiol.61.1.243. 10099689

[48] Lilburn TG, Cai H, Gu J, Zhou Z, Wang Y. 2014. Exploring systems affected by the heat shock response in Plasmodium falciparum via protein association networks. Int J Comput Biol Drug Des 7:369–383. doi:10.1504/IJCBDD.2014.066554. 25539848 PMC4492993

[49] Carter R, Miller LH. 1979. Evidence for environmental modulation of gametocytogenesis in Plasmodium falciparum in continuous culture. Bull World Health Organ 57:37–52. 397008 PMC2395706

[50] Liu Z, Miao J, Cui L. 2011. Gametocytogenesis in malaria parasite: commitment, development and regulation. Future Microbiol 6:1351–1369. doi:10.2217/fmb.11.108. 22082293 PMC5711484

[51] Buckling A, Ranford-Cartwright L, Miles A, Read AF. 1999. Chloroquine increases Plasmodium falciparum gametocytogenesis in vitro. Parasitology 118:339–346. doi:10.1017/S0031182099003960. 10340323

[52] Smalley M, Brown J. 1981. Plasmodium falciparum gametocytogenesis stimulated by lymphocytes and serum from infected Gambian children. Trans R Soc Trop Med Hyg 75:316–317. doi:10.1016/0035-9203(81)90348-5. 7029805

[53] Williams J. 1999. Stimulation of Plasmodium falciparum gametocytogenesis by conditioned medium from parasite cultures. Am J Trop Med Hyg 60:7–13. doi:10.4269/ajtmh.1999.60.7. 9988315

[54] Portugaliza HP, Miyazaki S, Geurten FJ, Pell C, Rosanas-Urgell A, Janse CJ, Cortés A. 2020. Exposure to artemisinin at the trophozoite stage increases sexual conversion rates in the malaria parasite Plasmodium falciparum. bioRxiv. https://www.biorxiv.org/content/10.1101/2020.06.15.151746v1. 10.7554/eLife.60058 PMC7577739 33084568

[55] Chaubey S, Grover M, Tatu U. 2014. Endoplasmic reticulum stress triggers gametocytogenesis in the malaria parasite. J Biol Chem 289:16662–16674. doi:10.1074/jbc.M114.551549. 24755215 PMC4059112

[56] Stephens JWW, Christophers SR. 1908. The practical study of malaria and other blood parasites. University Press of Liverpool, Liverpool, United Kingdom.

[57] Kafsack BFC, Rovira-Graells N, Clark TG, Bancells C, Crowley VM, Campino SG, Williams AE, Drought LG, Kwiatkowski DP, Baker DA, Cortés A, Llinás M. 2014. A transcriptional switch underlies commitment to sexual development in malaria parasites. Nature 507:248–252. doi:10.1038/nature12920. 24572369 PMC4040541

[58] Josling GA, Russell TJ, Venezia J, Orchard L, van Biljon R, Painter HJ, Llinás M. 2020. Dissecting the role of PfAP2-G in malaria gametocytogenesis. Nat Commun 11:1503. doi:10.1038/s41467-020-15026-0. 32198457 PMC7083873

[59] Josling GA, Williamson KC, Llinás M. 2018. Regulation of sexual commitment and gametocytogenesis in malaria parasites. Annu Rev Microbiol 72:501–519. doi:10.1146/annurev-micro-090817-062712. 29975590 PMC7164540

[60] Talman AM, Paul RE, Sokhna CS, Domarle O, Ariey F, Trape J-F, Robert V. 2004. Influence of chemotherapy on the Plasmodium gametocyte sex ratio of mice and humans. Am J Trop Med Hyg 71:739–744. doi:10.4269/ajtmh.2004.71.739. 15642963

[61] Martins RM, Macpherson CR, Claes A, Scheidig-Benatar C, Sakamoto H, Yam XY, Preiser P, Goel S, Wahlgren M, Sismeiro O, Coppée J-Y, Scherf A. 2017. An ApiAP2 member regulates expression of clonally variant genes of the human malaria parasite Plasmodium falciparum. Sci Rep 7:10. doi:10.1038/s41598-017-12578-y. 29070841 PMC5656681

[62] Painter HJ, Campbell TL, Llinás M. 2011. The Apicomplexan AP2 family: integral factors regulating Plasmodium development. Mol Biochem Parasitol 176:1–7. doi:10.1016/j.molbiopara.2010.11.014. 21126543 PMC3026892

[63] Modrzynska K, Pfander C, Chappell L, Yu L, Suarez C, Dundas K, Gomes AR, Goulding D, Rayner JC, Choudhary J, Billker O. 2017. A knockout screen of ApiAP2 genes reveals networks of interacting transcriptional regulators controlling the Plasmodium life cycle. Cell Host Microbe 21:11–22. doi:10.1016/j.chom.2016.12.003. 28081440 PMC5241200

[64] Silvestrini F, Lasonder E, Olivieri A, Camarda G, van Schaijk B, Sanchez M, Younis SY, Sauerwein R, Alano P. 2010. Protein export marks the early phase of gametocytogenesis of the human malaria parasite Plasmodium falciparum. Mol Cell Proteomics 9:1437–1448. doi:10.1074/mcp.M900479-MCP200. 20332084 PMC2938084

[65] Hermand P, Cicéron L, Pionneau C, Vaquero C, Combadière C, Deterre P. 2016. Plasmodium falciparum proteins involved in cytoadherence of infected erythrocytes to chemokine CX3CL1. Sci Rep 6:33786. doi:10.1038/srep33786. 27653778 PMC5031962

[66] Rowe JA, Claessens A, Corrigan RA, Arman M. 2009. Adhesion of Plasmodium falciparum-infected erythrocytes to human cells: molecular mechanisms and therapeutic implications. Expert Rev Mol Med 11:E16. doi:10.1017/S1462399409001082. 19467172 PMC2878476

[67] Moll K, Palmkvist M, Ch'ng J, Kiwuwa MS, Wahlgren M. 2015. Evasion of immunity to Plasmodium falciparum: rosettes of blood group A impair recognition of PfEMP1. PLoS One 10:e0145120. doi:10.1371/journal.pone.0145120. 26714011 PMC4694710

[68] Kyes SA, Kraemer SM, Smith JD. 2007. Antigenic variation in Plasmodium falciparum: gene organization and regulation of the var multigene family. Eukaryot Cell 6:1511–1520. doi:10.1128/EC.00173-07. 17644655 PMC2043368

[69] Rosenberg E, Ben-Shmuel A, Shalev O, Sinay R, Cowman A, Pollack Y. 2009. Differential, positional-dependent transcriptional response of antigenic variation (var) genes to biological stress in Plasmodium falciparum. PLoS One 4:e6991. doi:10.1371/journal.pone.0006991. 19730749 PMC2734987

[70] Hakimi M-A, Deitsch KW. 2007. Epigenetics in Apicomplexa: control of gene expression during cell cycle progression, differentiation and antigenic variation. Curr Opin Microbiol 10:357–362. doi:10.1016/j.mib.2007.07.005. 17719264

[71] Smith JD, Rowe JA, Higgins MK, Lavstsen T. 2013. Malaria's deadly grip: cytoadhesion of Plasmodium falciparum‐infected erythrocytes. Cell Microbiol 15:1976–1983. doi:10.1111/cmi.12183. 23957661 PMC3836831

[72] Alister GC, Mustaffa KMF, Pradeep RP. 2012. Cytoadherence and severe malaria. Malays J Med Sci 19:5. 22973133 PMC3431742

[73] Kaestli M, Cockburn IA, Cortés A, Baea K, Rowe JA, Beck H-P. 2006. Virulence of malaria is associated with differential expression of Plasmodium falciparum var gene subgroups in a case-control study. J Infect Dis 193:1567–1574. doi:10.1086/503776. 16652286 PMC2877257

[74] Rottmann M, Lavstsen T, Mugasa JP, Kaestli M, Jensen ATR, Müller D, Theander T, Beck H-P. 2006. Differential expression of var gene groups is associated with morbidity caused by Plasmodium falciparum infection in Tanzanian children. Infect Immun 74:3904–3911. doi:10.1128/IAI.02073-05. 16790763 PMC1489729

[75] Arnoldini M, Mostowy R, Bonhoeffer S, Ackermann M. 2012. Evolution of stress response in the face of unreliable environmental signals. PLoS Comput Biol 8:e1002627. doi:10.1371/journal.pcbi.1002627. 22916000 PMC3420966

[76] Dhar R, Sägesser R, Weikert C, Wagner A. 2013. Yeast adapts to a changing stressful environment by evolving cross-protection and anticipatory gene regulation. Mol Biol Evol 30:573–588. doi:10.1093/molbev/mss253. 23125229

[77] Young JW, Locke JC, Elowitz MB. 2013. Rate of environmental change determines stress response specificity. Proc Natl Acad Sci U S A 110:4140–4145. doi:10.1073/pnas.1213060110. 23407164 PMC3593889

[78] Walzer KA, Kubicki DM, Tang X, Chi J-TA. 2018. Single-cell analysis reveals distinct gene expression and heterogeneity in male and female Plasmodium falciparum gametocytes. mSphere 3:e00130-18. doi:10.1128/mSphere.00130-18. 29643077 PMC5909122

[79] Gravenor M, Kwiatkowski D. 1998. An analysis of the temperature effects of fever on the intra-host population dynamics of Plasmodium falciparum. Parasitology 117:97–105. doi:10.1017/S0031182098002893. 9778631

[80] Mbengue A, Bhattacharjee S, Pandharkar T, Liu H, Estiu G, Stahelin RV, Rizk SS, Njimoh DL, Ryan Y, Chotivanich K, Nguon C, Ghorbal M, Lopez-Rubio J-J, Pfrender M, Emrich S, Mohandas N, Dondorp AM, Wiest O, Haldar K. 2015. A molecular mechanism of artemisinin resistance in Plasmodium falciparum malaria. Nature 520:683–687. doi:10.1038/nature14412. 25874676 PMC4417027

[81] Li H, Handsaker B, Wysoker A, Fennell T, Ruan J, Homer N, Marth G, Abecasis G, Durbin R, 1000 Genome Project Data Processing Subgroup. 2009. The sequence alignment/map format and SAMtools. Bioinformatics 25:2078–2079. doi:10.1093/bioinformatics/btp352. 19505943 PMC2723002

[82] Trapnell C, Roberts A, Goff L, Pertea G, Kim D, Kelley DR, Pimentel H, Salzberg SL, Rinn JL, Pachter L. 2012. Differential gene and transcript expression analysis of RNA-seq experiments with TopHat and Cufflinks. Nat Protoc 7:562–578. doi:10.1038/nprot.2012.016. 22383036 PMC3334321

[83] Bahl A, Brunk B, Crabtree J, Fraunholz MJ, Gajria B, Grant GR, Ginsburg H, Gupta D, Kissinger JC, Labo P. 2003. PlasmoDB: the Plasmodium genome resource. A database integrating experimental and computational data. Nucleic Acids Res 31:212–215. doi:10.1093/nar/gkg081. 12519984 PMC165528

