# Supplementary material for: Single-Cell RNA Sequencing Reveals Cellular Heterogeneity and Stage Transition under Temperature Stress in Synchronized Plasmodium falciparum Cells
Source: Microbiol Spectr. 2021 Jul 7;9(1):10.1128/spectrum.00008-21. doi: 10.1128/spectrum.00008-21 (PMC8552519; doi:10.1128/spectrum.00008-21)

Single-cell RNA sequencing reveals cellular heterogeneity and stage transition during stress condition in synchronized *Plasmodium falciparum*

Mukul Rawat[1], Ashish Srivastava[1†], Shreya Johri[2], Ishaan Gupta[2§] and Krishanpal Karmodia[1§*]

[1]Department of Biology, Indian Institute of Science Education and Research, Dr. Homi Bhabha Road, Pashan, Pune 411008, Maharashtra, India
[2]Department of Biochemical Engineering and Biotechnology, Indian Institute of Technology Delhi New Delhi, 110016, India

§Corresponding authors
Correspondence to: ishaan@iitd.ac.in; krish@iiserpune.ac.in
*Lead contact: krish@iiserpune.ac.in
[†]Deceased on 06th April, 2019

Running title: ScRNA-seq under stress condition in *P. falciparum*

Keywords: Malaria; *Plasmodium falciparum*; Single-cell RNA sequencing; Stress response; Antigenic variation; Transportation

Supplementary Information

Table S1: Markers genes which are expressed at a higher level in each cluster are provided as an excel sheet.

Table S2: List of genes belonging to different modules and their gene ontology and list of genes deregulated across clusters in single-cell RNA sequencing.

Table S3: Expression level of all known gametocyte regulators and markers across different clusters

Table S4: Primers used for RT-qPCR.

Table S5: Gene list used in this study; (A) stress-responsive genes, (B) gametocyte regulators and markers, (C) clonally variant multicopy gene families (*var, rifin* and *stevor*), (D) genes exhibited highest variation during control and temperature treatment.

Supplementary Figure legends

Figure S1: (A) Flow cytometry evaluation of control and temperature treated parasites using the Annexin V-FITC (fluorescein isothiocyanate) staining. Cell death estimation in control and temperature treated parasites is 0.41% and 0.54% of total cells, respectively. (B) Representative tSNE plots showing the expression of PF3D7_0532600 (*Plasmodium* exported protein) and PF3D7_0511200 (stearoyl-CoA desaturase) in control and treatment condition. (C) Cluster 8 is a unique cluster present only during temperature treatment. To confirm if cluster 8 represents a novel cluster or dead parasites, we analysed mitochondrial gene expression in all the clusters during temperature treated condition. Percentage of mitochondrial gene expression per cell in Cluster 8 is comparable to all other clusters indicating that it does not represent dead parasites. (D) Random down sampling was performed to cluster equal numbers of cells from control and treatment samples. Bar graph represents the frequency of occurrence of Cluster 8 (unique to treated sample) and number of cells identified in the unique cluster. Down sampling the number of cells sequenced does not leads to disappearance of Cluster 8. (E) Similar numbers of cells from control and temperature treatment were co-clustered to identify the frequency with Cluster 8 is found only during temperature treatment

making it a unique cluster. Ninety-seven out of hundred times, we found that the original cells of the "new cluster" in temperature treatment made a unique cluster with 100% (denoted by 1.0 on y-axis) treated cells, with a cluster size between 80-100% (0.8-1.0 on x-axis) of the total number of cells in the original cluster.

Figure S2: (A) Table showing gene ontology terms enriched in each cluster for cluster specific marker genes and deregulated genes under temperature treatment. (B) Average RNA molecules expressed per cell is plotted for cells under control and temperature treatment. Downregulation in the number of RNA molecules was observed during temperature treatment. Significance was determined using a paired t-test. * $p < 0.05$; *** $p < 0.005$. (C) Average numbers of RNA molecules per cell for each cluster under control and temperature treatment. Significance was determined using a paired t test. * $p < 0.05$; *** $p < 0.005$ (D) Estimation of the level of RNA molecules by labelling RNA using an RNA specific dye SYTO RNASelect in control and temperature treated parasites. Flow cytometry analysis of the temperature treated parasites shows overall downregulation in the number of RNA molecules. (E) A representative confocal image shows down regulation in the fluorescence intensity of the SYTO RNASelect after temperature treatment indicating downregulation in the number of RNA molecules in the parasite. (F) Coefficients of variation under control condition are sorted in ascending order and coefficients of variation under temperature treatment are examined over them. The Fligner-Killeen Test for equal coefficients of variation suggest significant variation between control and temperature treatment (CV, 128.19 and 136.32 for control and temperature treatment, respectively; p value =2.08e-5). (G) Bar plot showing the gene ontology terms such as gametocytogenesis, chaperon activity and maintenance of cellular homeostasis which show maximum variation under temperature treatment.

Figure S3: (A) Bubble plot shows relative expression of stress-responsive genes across clusters in control and during temperature treatment. Bubble size is proportional to percentage of cells expressing a gene, and color intensity is proportional to average scaled gene expression within a cluster. (B) Bulk RNA sequencing also shows global upregulation in expression of heat shock proteins after temperature treatment. Significance was determined using a paired t test. * $p < 0.05$; *** $p < 0.005$. (C) Bulk RNA sequencing shows global downregulation in the expression of ubiquitin proteasome proteins after the temperature treatment. Significance was determined using a paired t test. * $p < 0.05$; *** $p < 0.005$

Figure S4: (A) Bar plot showing the upregulation of various *var* genes during temperature treatment as measured by scRNA-sequencing. (B) Bar blot showing the upregulation of various *var* genes during temperature treatment as measured by bulk RNA sequencing.

Figure S5: (A and B) Sanity check plots of number genes (nFeature_RNA), number of RNA molecules (nCount_RNA) and percentage of mitochondrial genes (genes beginning with "mal" in the annotation file) across individual cells in the dataset for both control and temperature treatment sample respectively. (C and D) Sanity check plots to see any deviations from the expected relationship between number of RNA molecules (nCount_RNA) and percentage of mitochondrial gene expression (percent.mt); expected to be not correlated as shown; and number of genes (nFeature_RNA); expected to be linear because the deeper a cell is sequenced the more number of genes should be detected per cell. (E, F) JackStraw procedure was implemented to determine the dimensionality of the datasets. 10 significant principal components (PCs) with strong enrichment and low P-value were identified. Elbow plots 6 (ranking of principal components based on the percentage of variance) of 20 principal components (PC) for the control and the treated sample show that the percentage variation explained per PC flattened around PC10. Hence, only 10 PCs were taken for downstream analysis like cell clustering. t-Distributed Stochastic Neighbor Embedding (t-SNE) projections of gene expression for

control as well as treatment data set using nearest neighbour graph based clustering followed by Louvain algorithm suggested 8 clusters across both data sets. Both the datasets were combined to explore clusters which are common between the two conditions.

Supplementary Figure S1

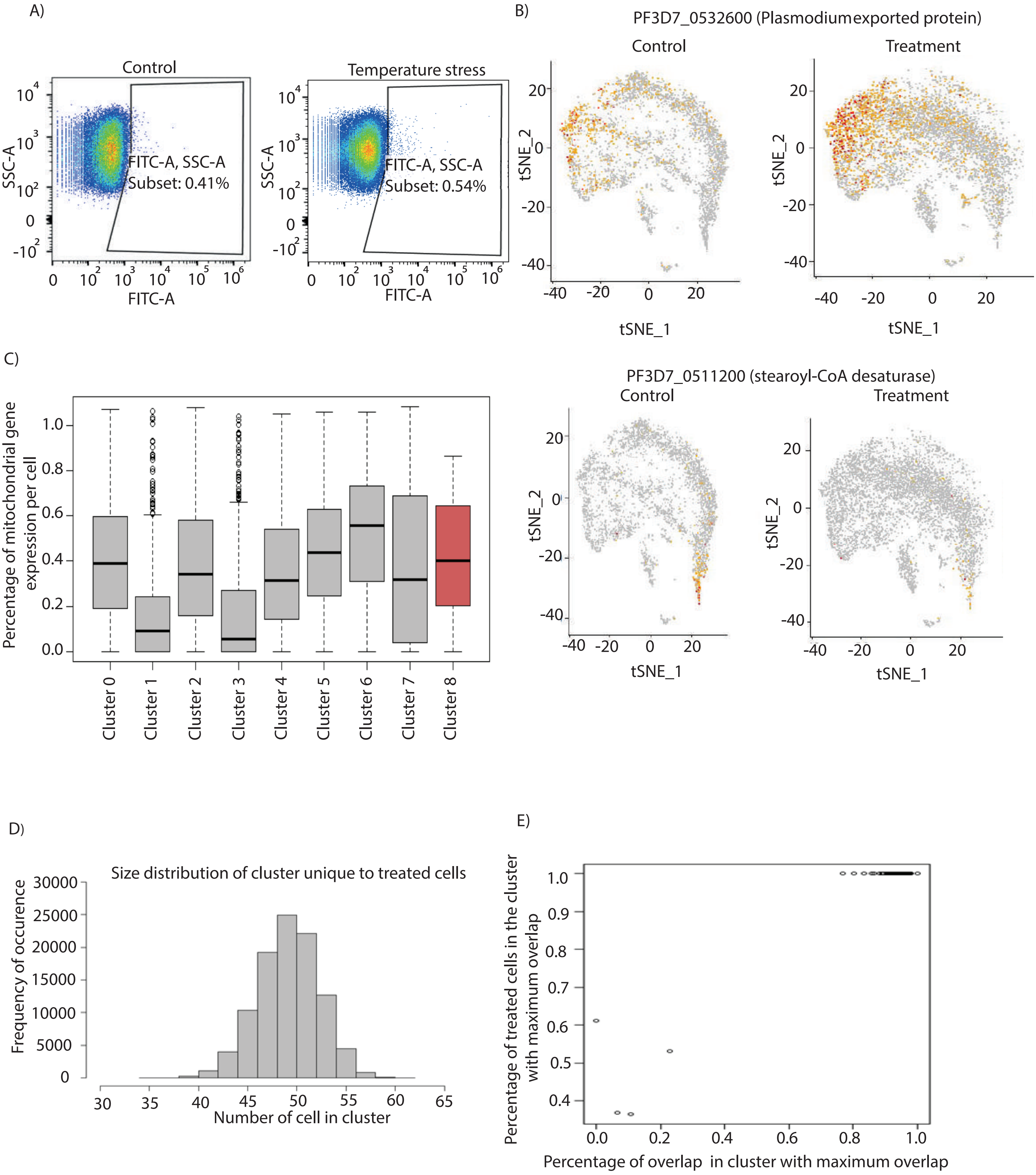

Supplmentary Figure S2

A)

Cluster	Cluster specific marker gene<br>Gene ontology terms (p-value)	Cluster specific upregulated genes<br>Gene ontology terms (p-value)	Cluster specific downregulated genes<br>Gene ontology terms (p-value)
0	purine ribonucleoside salvage (1.99e-3)	sister chromatid segregation (0.0152)	C-terminal protein methylation (0.00575)
1	translation (2.28e-8)<br>protein metabolic process (1.43e-5)	multi-organism transport (1.34e-04)	glutamine metabolic process (9.06e-04)
2	gluconeogenesis (4.21e-4)<br>glucose metabolic process (6.29e-4)	neutral lipid metabolic process (4.21e-03)	nonribosomal peptide biosynthetic process (0.0102)
3	translocation of molecules into host (7.15e-3)<br>multi-organism transport (7.15e-3)	cell-cell adhesion	interaction with host (7.94e-04)
4	glutathione metabolic process (4.42e-3)<br>sulfur compound metabolic process (3.60e-2)	heme catabolic process (3.98e-03)	GTP metabolic process (8.83e-03)
5	response to drug (1.00e-3)<br>response to chemical (8.02e-3)	DNA conformation change (5.91e-05)	microtubule nucleation (1.76e-02)
6	immunoglobulin production (1.20e-5)<br>regulation of immune response (5.41e-5)	protein dephosphorylation (2.11e-02)	microtubule-based movement (1.87e-03)
7	cellular lipid metabolic process (1.18e-2)<br>nucleobase metabolic process (2.95e-3)	actin nucleation (6.25e-04)	locomotion (4.35e-03)

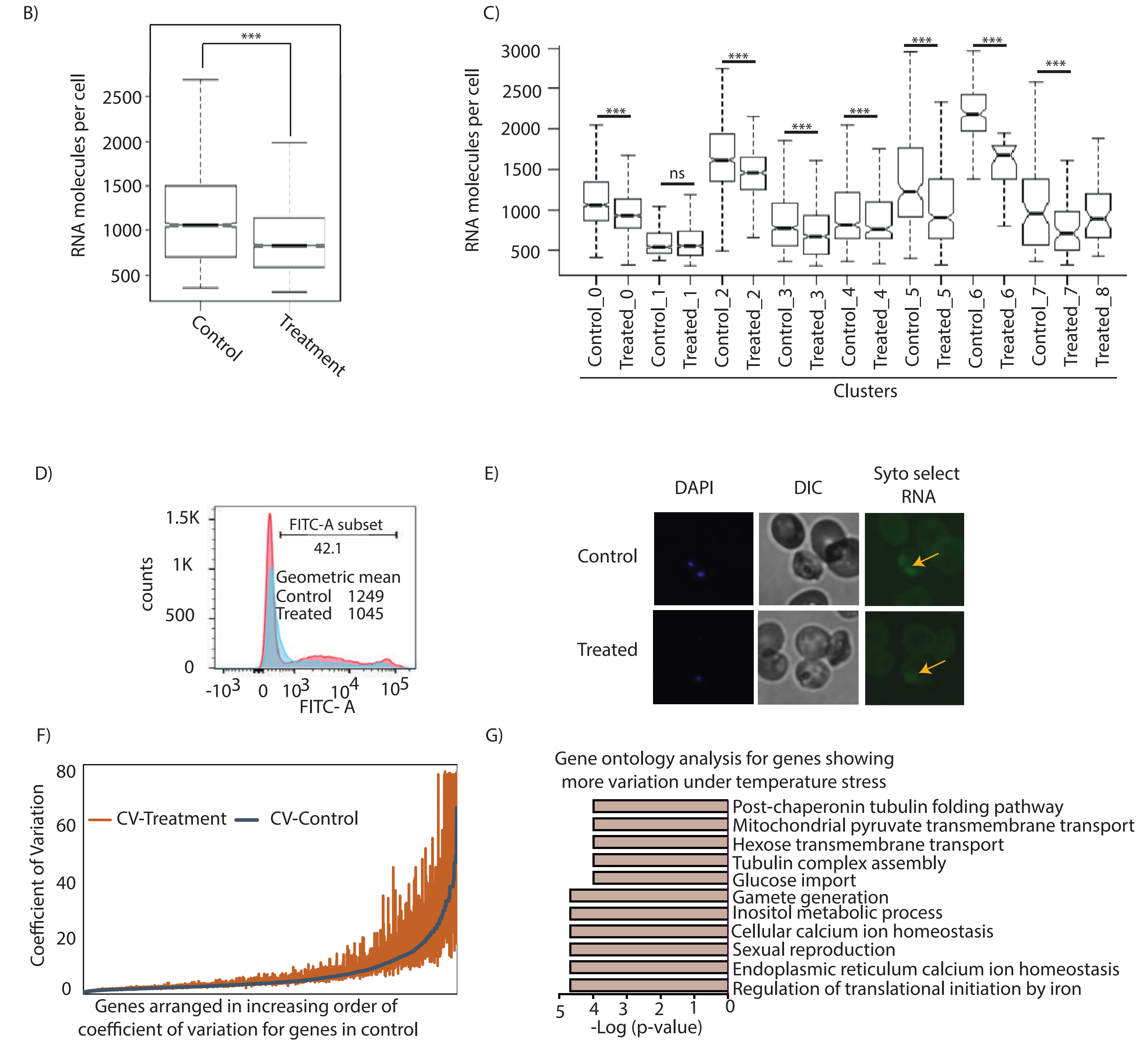

Supplementary Figure S3

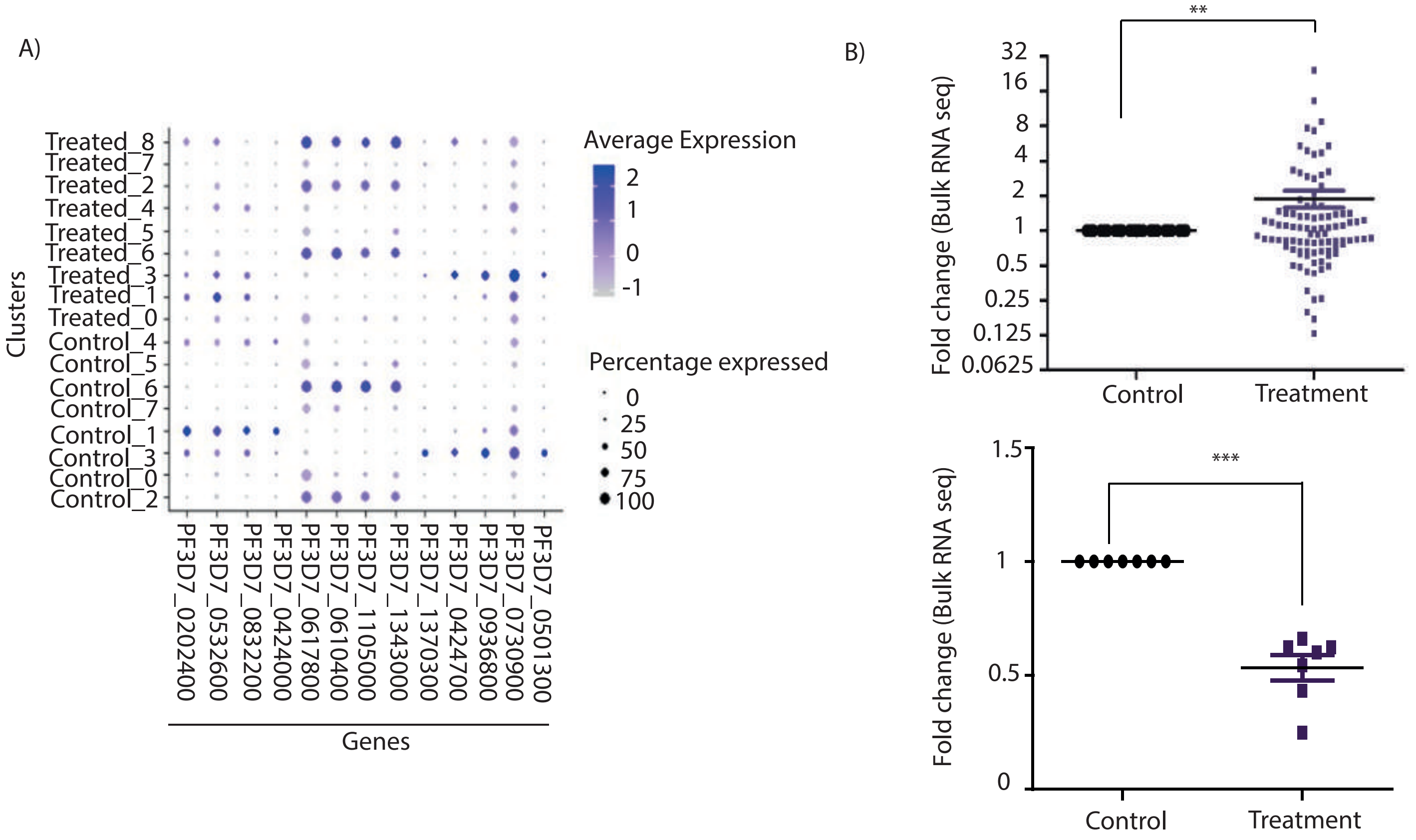

Supplementary Figure S4

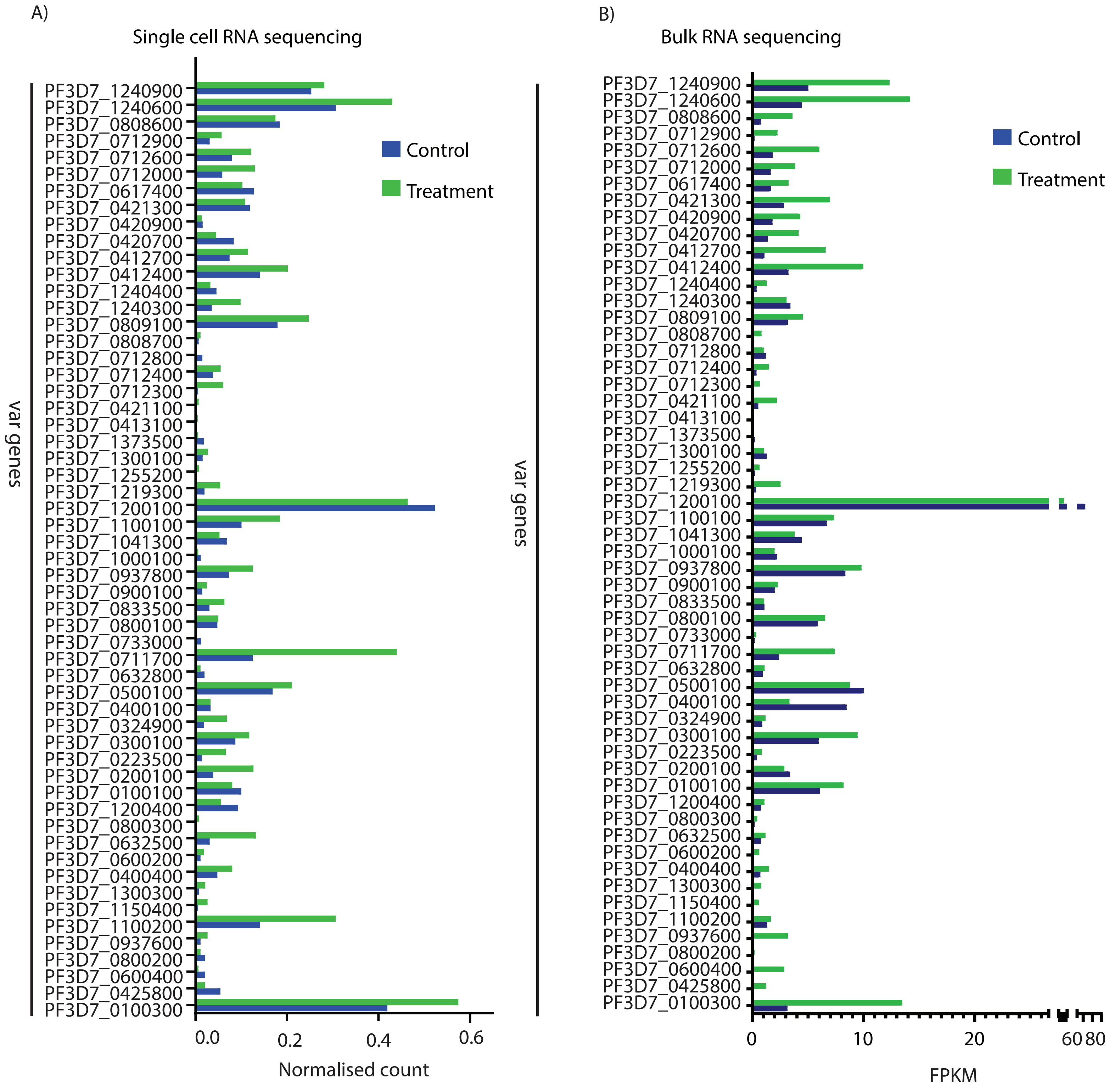

Supplmentary Figure S5

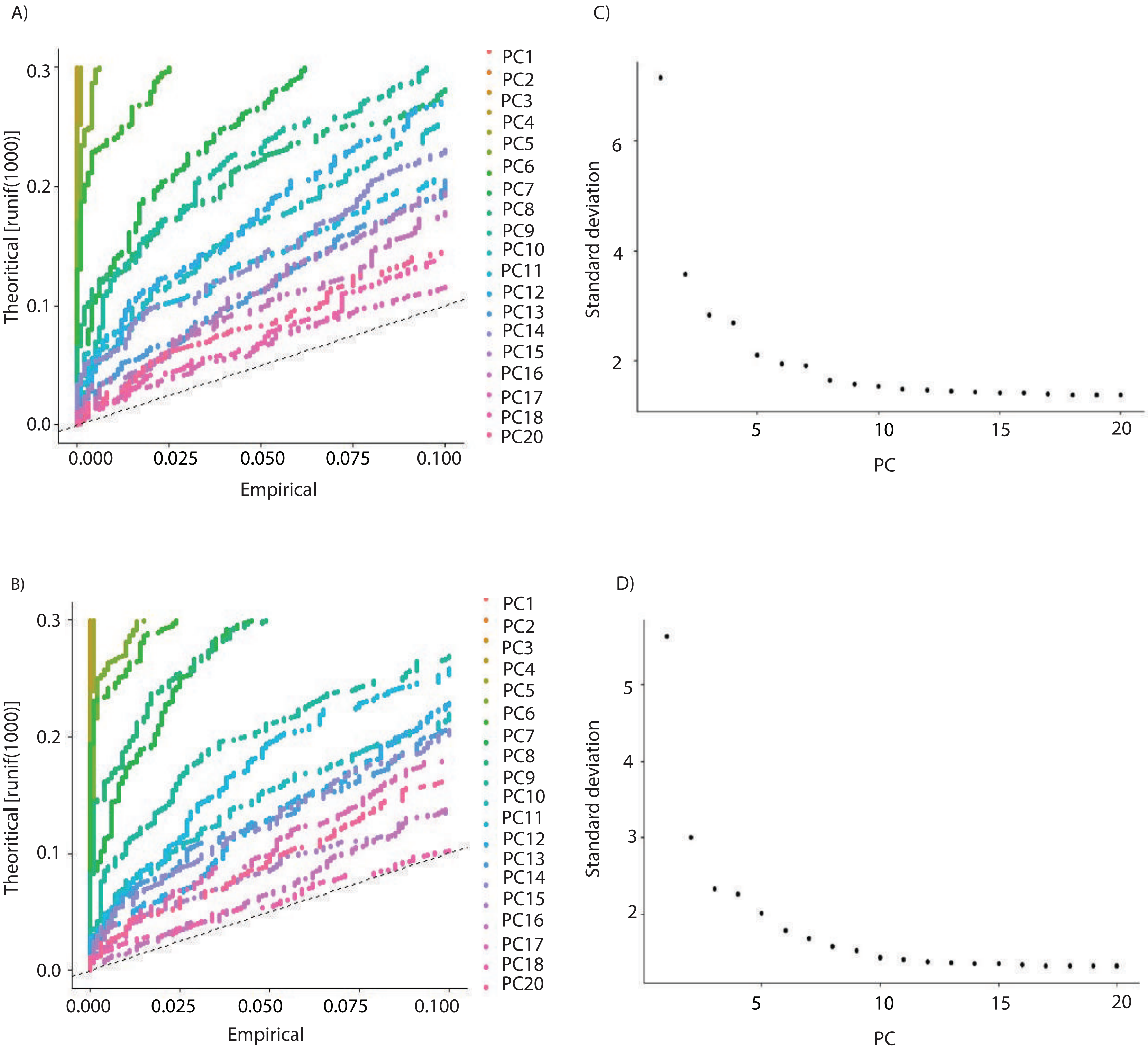

Supplement: SUPPLEMENTAL FILE 1 — Supplemental material. Download SPECTRUM00008-21_Supp_1_seq9.pdf, PDF file, 1.6 MB [file spectrum00008-21_supp_1_seq9.pdf]
